# Synthesis of new zwitterionic surfactants and investigation of their surface active and thermodynamic properties

**DOI:** 10.1038/s41598-025-97814-6

**Published:** 2025-05-06

**Authors:** Ahmed S. Mansour, M. M. Abo-Aly, S. A. Rizk, Abdellatif M. M. Abd El Rahman, N. F. Ghaly, A. A. Ragab, A. M. Alsabagh

**Affiliations:** 1 Application Department, Egyptian Petroleum Research Institute, Cairo, Egypt; 2 Chemistry Department, Faculty of Science, Ain Shams University, Cairo, Egypt

**Keywords:** Zwitterionic surfactant, Surface tension, Interfacial tension, Thermodynamic properties and oil production, Chemistry, Materials science

## Abstract

This study focused on the synthesis of six bio-based zwitterionic surfactants derived from oleic acid to assess their applicability in different petroleum fields. The final bi-zwitterionic surfactants were synthesized from oleic acid, utilizing the double bond and carboxylic group. Friedel–Crafts alkylation, sulfonation, chlorination, amidation, and quaternization were performed to synthesize six bi-zwitterionic surfactants. The bi-quaternary surfactants derived from benzene are represented by the general formula Bi Q 10, BOAS (Amide), with the symbols BE, BP, and BPh. In contrast, those derived from naphthalene are represented by Bi Q 10, NOAS (Amide), with the symbols NE, NP, and NPh. The structures of these surfactants were confirmed using FT-IR and H^1^-NMR techniques. The surface activity and thermodynamic properties of the synthesized surfactants were analyzed through surface tension measurements conducted at various temperatures (30, 40, 50, and 60°C). Additionally, **CMC, γ**_**CMC**_**, π**_**CMC**_**, Γ**_**max**_**, A**_**min,**_ and **Pc**_**20**_ were measured. The thermodynamic variables for micellization and adsorption were also measured. The structural effect of the obtained surfactants was assessed. The maximum value of the structural effect was 4.33 KJmol^-1^, corresponding to **BE**. The results indicated that the negative values of ΔG_ads_ were greater than the negative values of ΔG_mic_, indicating that these surfactants are absorbed in the interface prior to the formation of micelles. The more negative values of ΔG_ads_ suggest that these surfactants are strongly adsorbed onto solid particles, such as sands and rocks, indicating their potential utilization in oil production in different petroleum fields.

## Introduction

Surfactants are extensively applied in various fields, including household detergents and industrial applications. Applications encompass well stimulation, drilling fluid, swelling inhibition, filter cake removal, fracturing, corrosion prevention, and EOR. The surfactant’s ability to alter the reservoir’s wetting characteristic to a water-wet state is also an essential factor in surfactant selection for oil production in different petroleum fields. Surfactants can be classified as cationic, anionic, nonionic, and zwitterionic based on the type of their polar moiety.

In recent decades, the reservoir oil extraction rate has decreased, highlighting the need for innovative recovery technologies^1^. The application of surfactants in oil production in different petroleum fields is a highly effective method for augmenting oil extraction from low-pressure reservoirs. The surfactant chemical flooding is an effective method used to mobilize the majority of residual oil trapped in reservoirs.

One of the objectives of surfactant injection into the reservoir is to enhance the oil extraction by mitigating asphaltene precipitation^2^. In addition to their excellent surface activity and wettability, surfactants must also be biodegradable and non-toxic^3^.

The characteristics of surface activity and thermodynamics offer insights into the organization, adsorption, and micellization of surfactant molecules across two phases, as well as the reduction of surface tension^4,5^. Gibbs isotherms serve as a tool for analyzing the micellization and adsorption processes at the interface^6–11^. Micellization and adsorption processes are critical for elucidating factors that affect CMC values, such as the structural effect^12,13^. Micelles form when the surfactant concentration nears the critical micelle concentration (CMC), which is affected by reservoir parameters, including temperature, pressure, and formation water salinity.

The adsorption of surfactants on reservoir rocks presents a significant challenge requiring extensive research before their application in specific reservoirs^14^.

The selection of surfactants to be applied in a reservoir depends on several aspects, including the classification of surfactants, reservoir type, pH, salinity, and thermal conditions present within the reservoir. Surfactants are classified as anionic, cationic, non-ionic, and zwitterionic, based on the electrical charge associated with the hydrophilic head group of the surfactants. Anionic surfactants possess negatively charged hydrophilic head groups, such as carbonate, sulfate, and sulfonate ions, while cationic surfactants are characterized by positively charged hydrophilic head groups, including ammonium and imidazolium ions. Non-ionic surfactants exhibit a neutral charge, and their surface activity is attributed to the ethylene oxide or propylene oxide moieties incorporated within their molecular framework. Zwitterionic surfactants represent a distinct category that contains both positively and negatively charged entities within their hydrophilic head. The surfactant type serves as a critical criterion for selection according to specific reservoir types, as anionic surfactants have demonstrated incompatibility with carbonate rocks, and cationic surfactants are unsuitable for sandstone reservoirs. Non-ionic surfactants exhibit superior applicability in high salinity reservoirs; however, their predominant mechanism for oil recovery is related to wettability alteration^15^. The presence of amphoteric charges in zwitterionic surfactants which neutralize each other under normal conditions make them particularly adaptable for utilization in both sandstone and carbonate reservoirs, and their overall neutral charge enhances compatibility for application in high salinity environments^16^. Zwitterionic surfactants exhibit superior properties, including biodegradability, salt and high-temperature resistance, favorable water solubility, adequate lipophilicity, and good synergistic effect with certain nonionic and anionic surfactants. These characteristics contribute to their efficacy in oil production in different petroleum fields^17^. Shafek Samir H., et al. (2023) synthesized three zwitterionic surfactants based on azomethine with different hydrophobic chains labeled ZWSO, ZWSD, and ZWSH used for improving C-steel protection and they concluded that, the presence of azomethine group, electrons, and heteroatoms in the zwitterionic surfactant’s amphipathic structure helped to improve C-steel protection^18^. Gbadamosi, Afeez, et al. (2024) examined six in-house developed zwitterionic surfactants (betaine-type surfactants) that demonstrate significant thermal and salinity tolerance, focusing on their behavior at the oleic–aqueous interface for applications in oil recovery. They found that based on the surfactant headgroup, the magnitude of surfactant adsorption was in the order of sulfobetaine > hydroxysulfobetaine > carboxybetaine zwitterionic surfactant, respectively^19^. Zhang, Youhua, et al. (2024) designed an environmentally friendly zwitterionic fluorocarbon surfactant (PFSC) by shortening fluorocarbon chains and covalent bonding them with the zwitterionic groups to demonstrate their effect on the emulsion polymerization of fluoropolymers. The results demonstrated that the fluorinated surfactant exhibits excellent emulsification effects and can serve as an alternative to perfluorooctanoic acid^20^. Liu, Fang‐Hui, et al. (2023) developed a bio-based zwitterionic surfactant, N-phenylpropanaldehyde epoxy acetal octadecanoicamido propyl-N, N-dimethyl hydroxy sulfonate (PADS), derived from methyl oleate, for application in tertiary oil recovery. For synthesis of PADS, methyl oleate was first epoxidized to create a new reactive site. Acetalization, a more environmentally friendly technique, was then used to add the benzene ring to hydrophobic chains of surfactants. The results demonstrated that PADS has outstanding interfacial activity and potential for low-dose use. It could lower the interfacial tension between crude oil and simulated formation water to ultra-low in a broad concentration range under alkali-free environment^21^.

BAGHERSAEI, Shirin, et al.(2024), synthesized three zwitterionic compounds and characterized them using FT-IR, NMR spectroscopy, thermogravimetric, and differential thermal analysis. These compounds were 2-(1-methyl-1H-imidazol-3-ium-3-yl)butane-1-sulfonate (MIBS), 4-(pyridin-1-ium-1-yl) butane-1-sulfonate (PBS), and 4-(tributylammonio)butane-1-sulfonate (TBBS). different methods were used to examine these zwitterions’ ability to decrease asphaltene precipitation. In comparison to the other two zwitterions, the MIBS zwitterion, which contains an imidazole cation, was the most effective for dispersing asphaltene aggregates^22^.

Generally, surfactants affect oil extraction processes via four primary mechanisms: interfacial tension reduction, wettability alteration, foam generation, and emulsification^17^. There is a relationship between the structure of the surfactants and their function in the application field. The molecular structure of surfactants significantly affects thermodynamic behavior, especially in EOR applications. The long alkyl chain length could effectively alter rock wettability from oil-wet to more water-wet and reduce the IFT to a lower range^23,24^. Branched hydrocarbon surfactant can form a well-stabilized microemulsion due to the high lipophilicity, which is affected by the amount and the type of branching^25,26^. The addition of ethylene oxide or propylene oxide to the chemical structure of surfactant leads to the formation of low-viscosity microemulsion and ultra-low IFT due to the formation of hydrogen bonding between these groups and water. This facilitates the adsorption of surfactants at the water/oil interface and mitigates the salting-out effect, resulting in reduced IFT^27,28^. Surfactants containing sulphonate or carboxylate units produced high oil recovery for carbonate and sandstone reservoirs^27,29^.

This paper represents a series of bi-zwitterionic surfactants derived from oleic acid: BE, BP, BPh, NE, NP, and NPh. Their chemical structure is confirmed by H^1^-NMR and FT-IR^30^. Zwitterionic surfactants are compounds that contain a cation and an anion in different atoms of the same molecule, making them electrically neutral and giving them the opportunity to behave as acids or bases (donor or acceptor) according to the characteristics of the environment in which they are found. As a result, these molecules behave as smart wettability modifiers that can respond efficiently depending on the characteristics of their specific environments.

Surface activity and thermodynamic characteristics can be calculated using surface and interfacial tension calculations. The adsorption parameters of surfactants can be utilized to characterize the efficency of these surfactants to be used dring the oil production process.

## Materials and methods

### Materials

All chemicals utilized were of analytical grade, including AlCl_3_ (99%, central drug house , New Dlhi, India), benzene (99%, BioChem , Egypt), hydrochloric acid (37%, ADWIC, Egypt), thionyl chloride (97%, Merck , Germany), acetone (99%, EPRI , Egypt), N,N-dimethyl-1,3-propanediamine (99%, Alpha Aeser, Germany), methanol (99.5%, BioChem, Egypt), sodium chloroacetate (98%, ThermoFisher, Germany), ethanol (99%, Sigma-Aldrich, USA ), ethyl acetate (99%, EPRI, Egypt), N,N-dimethyl-1,2-ethylenediamine (96%, ThermoFisher, Germany), N,N-dimethyl-1,4-phenylenediamine (97%, ThermoFisher, Germany), Oleum acid (29%, PubChem, USA), Sodium hydroxide (96%, ADWIC, Egypt) and Oleic acid (SISCO Research Laboratories, India) with saponification value determined as 196–204 mg KOH/g.

### Synthesis of the bi-zwitterionic surfactants

#### Synthesis of BE, BP & BPh based on benzene as in Fig. 1

**Fig. 1:**
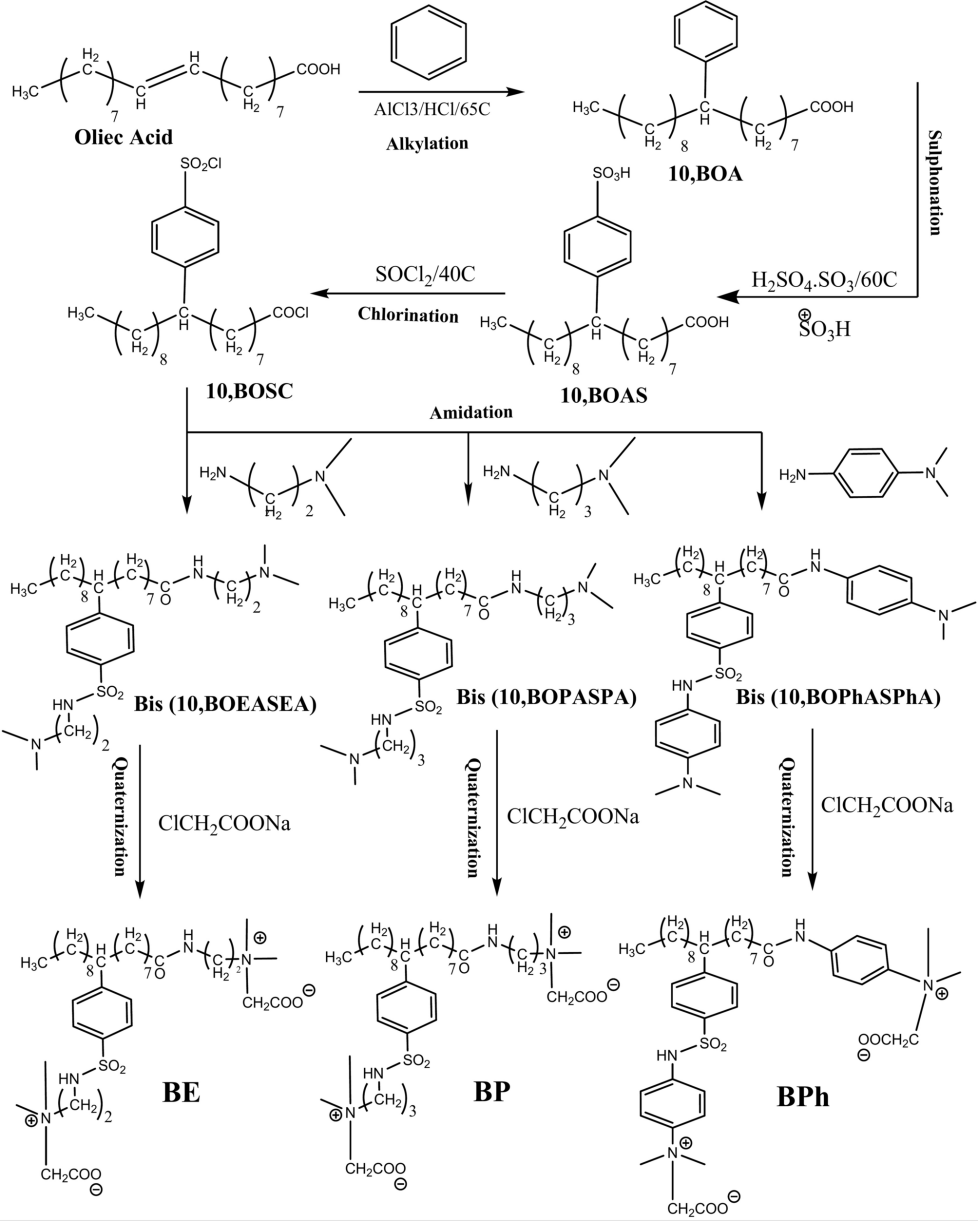
Synthesis of BE, BP & BPh based on benzene.

(a) Alkylation of benzene by oleic acid.

Oleic acid and AlCl_3_ were added in a 1:1 molar ratio in a dried round-bottom flask under vigorous stirring. Benzene was subsequently added, and the mixture was refluxed for six hours at 65 °C. Anhydrous CaCl_2_ was positioned in a dried tube above the condenser’s top. Following the alkylation reaction, the product was washed thrice with 15 mL of 1.0 M hydrochloric acid. The residual benzene was then recycled via vacuum distillation, yielding the compound designated as 10-benzene oleic acid **(10, BOA),** with a yield of 90%.

(b) Sulphonation of (10, BOA).

A solution of **(10, BOA)** in trichloromethane was added to a dried flask. Oleum, 30% SO_3_(H_2_SO_4_.SO_3_), was added dropwise to the solution while maintaining vigorous stirring for 30 min at 60 °C until obtaining a viscous brown liquid. The mixture was vacuum distilled off to remove the trichloromethane and the residual 30% SO_3_ (H_2_SO_4_.SO_3_), resulting in the desired BOAS product (liquid) and the removal of the unreacted residue of BOA. The product was named 10, benzene oleic acid sulphonate **(10, BOAS)**, and yielded 88%.

(c) Chlorination of (10, BOAS).

A solution of **(10, BOAS)** in trichloromethane was added dropwise to a dried flask, followed by the dropwise addition of thionyl chloride (SOCl_2_) to the reactant. The mixture was subsequently vacuum-distilled to eliminate the solvent and reactants. The product named 10, benzene olyl sulphonyl chloride **(10, BOSC)**, had a product yield of 87%.

(d) Amidation of (10, BOSC).

In this step, the bis amide of **(10, BOSC)** was produced by separately amidating it with N,N-dimethyl-1,2-ethylenediamine, N,N-dimethyl-1,3-propanediamine, and N,N-dimethyl-1,3-phynylenediamine.

The reactant was dissolved in 10 ml acetone, and three types of diamines were slowly added separatly at 0 °C. The reaction mixture was then heated to 40°C and stirred for 2 h. Vacuum distillation of the reaction mixture was conducted to obtain pure bis amide, specifically bis 10, benzene olyl amide sulphonyl amide, represented by the general formula bis **(10, BOASA)**. This process produced three products corresponding to three different amines, with yields of 83, 82, and 85%, respectively.

(e) Quaternization of bis (10, BOASA).

The amidation process yielded three products, which were subsequently quaternized with sodium chloroacetate at a molar ratio of 1:1.25 in a solvent mixture of methanol and water at a volume ratio of 1:1. The reactants were refluxed at 75 °C for 12 h; vacuum distillation was employed to eliminate solvents and unreactants. The final product was dissolved in ethanol and then filtered. Recrystallization in ethylacetate was carried out to obtain the final pure quaternized products **BE, BP, and BPh**, yielding 85, 86, and 83%, respectively.

#### Synthesis of NE, NP &NPh based on naphthalene as in Fig. 2

**Fig. 2:**
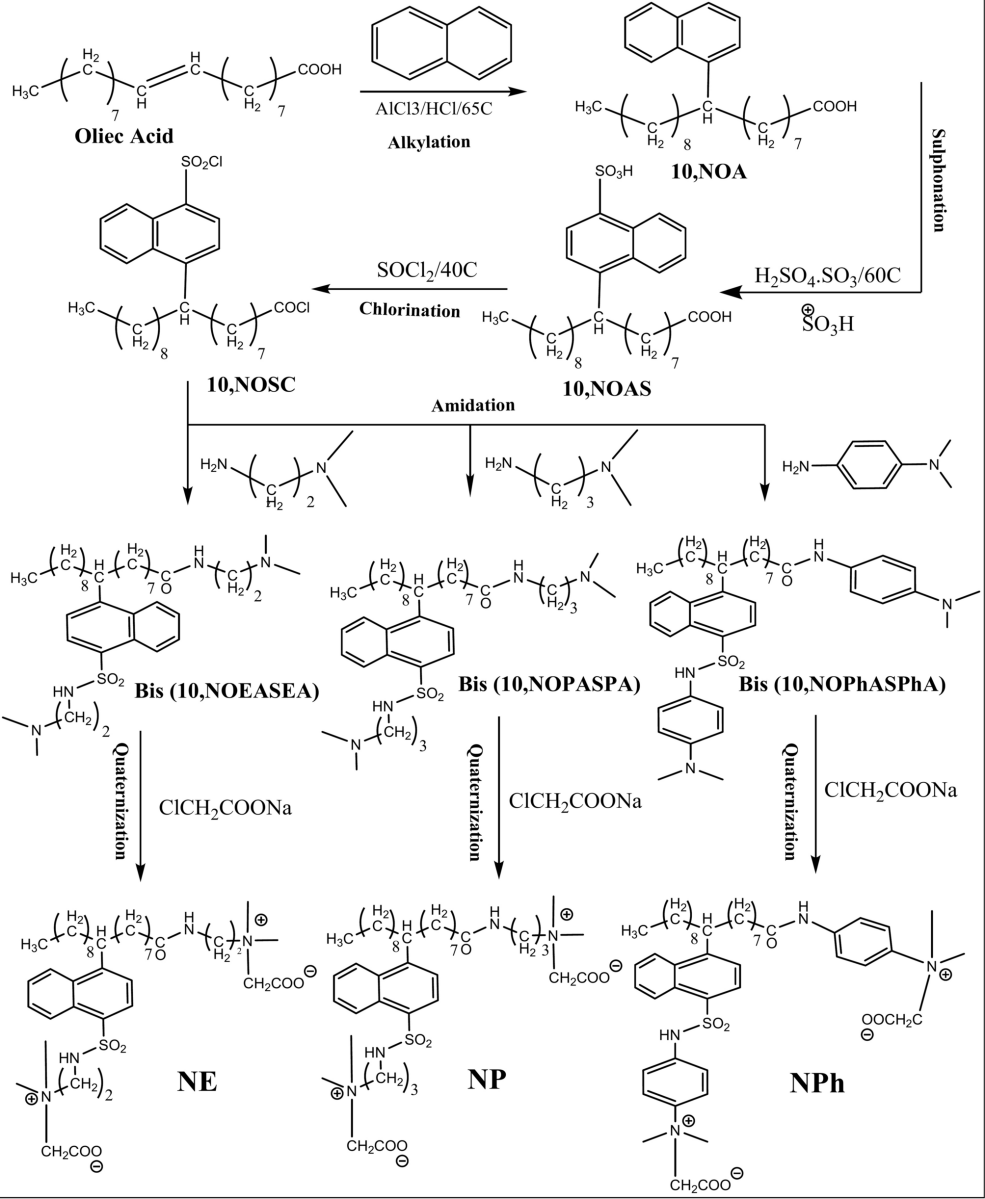
Synthesis of NE, NP & NPh based on naphthalene.

The final steps were repeated, substituting the benzene ring with naphthalene, resulting in the second series of surfactants NE, NP, and NPh, with yields of 86%, 84%, and 82%, respectively.

### Chemical structure characterization

#### Fourier transform infrared spectroscopy (FT-IR)

FTIR was used to elucidate the functional characteristics of the synthesized surfactants, providing detailed insights into their molecular structure. The IR transmission spectroscopic analysis was conducted at room temperature using a JASCO Nicolet IS-10 spectrometer, utilizing the K-Br disc method.

#### ^1^H-nuclear magnetic resonance

The (^1^H-NMR) spectroscopy of the prepared surfactants was recorded using a Bruker Avance 400 spectrometer (400 MHz) in D_2_O at room temperature, and tetramethylsilane (TMS) was used as a reference.

### Surface tension measurements

Using the Du Nouy ring technique, the surface tension values at varying temperatures ( 30, 40, 50, and 60 °C) at the air–water interface with and without surfactants (at various concentrations) were determined by using Kruss Gm BH k Easy Dyne tensiometer. Measurements were done three times and we took the main of the three values. Following each reading, the platinum ring was cleaned using a mixture of ethyl alcohol and acetone and then dried with a flame prior to the subsequent measurement. The surface active and thermodynamic properties were evaluated using the obtained results.

### Interfacial tension measurements

The interfacial tension between the studied surfactants and crude oil has been analyzed. The properties of crude oil were measured, yielding an API gravity of 13.09 at 60 °C, asphaltenes content of 9.46%, saturates content of 39.55%, resins content of 14.95%, aromatics content of 36.04%, and a density of 0.9777 at 15 °C. The IFT data was obtained utilizing a spinning drop tensiometer. The capillary tube containing the surfactant solution was rotated at 3500 rpm after the introducting of a crude oil drop into it. When comparing crude oil to formation water, the measured blank value for interfacial tension was 19.45 mNm^-1^. Surfactants can be utilized to be used during the oil production in different petroleum fields .

## Results and discussion

### Chemical structure confirmation of the prepared surfactants

#### Fourier transform infrared spectroscopy (FT-IR)

**Fig. 3:**
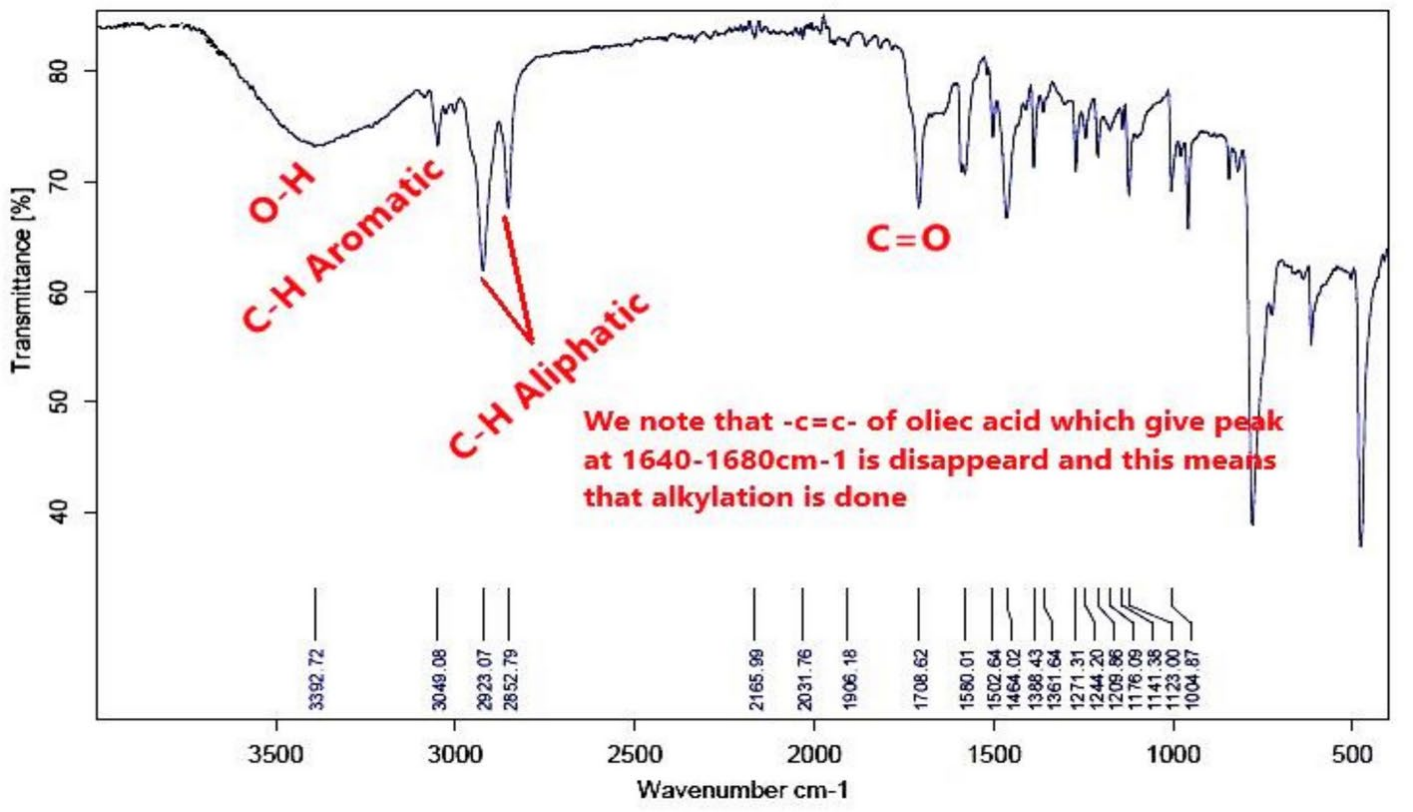
FT-IR of Alkylated Naphthalene by Oleic Acid.

**Fig. 4:**
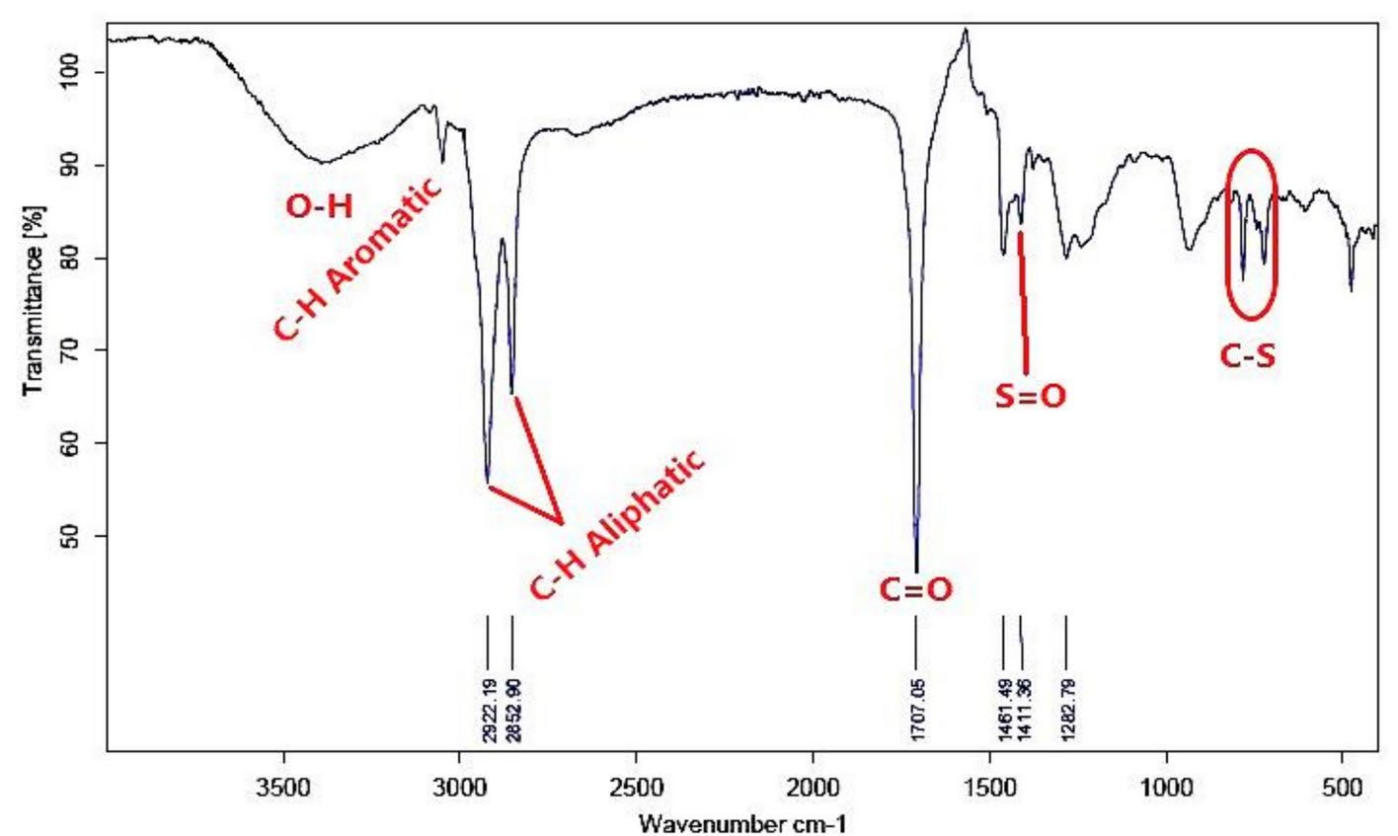
FT-IR of Sulphonation Step of 10, NOA.

**Fig. 5:**
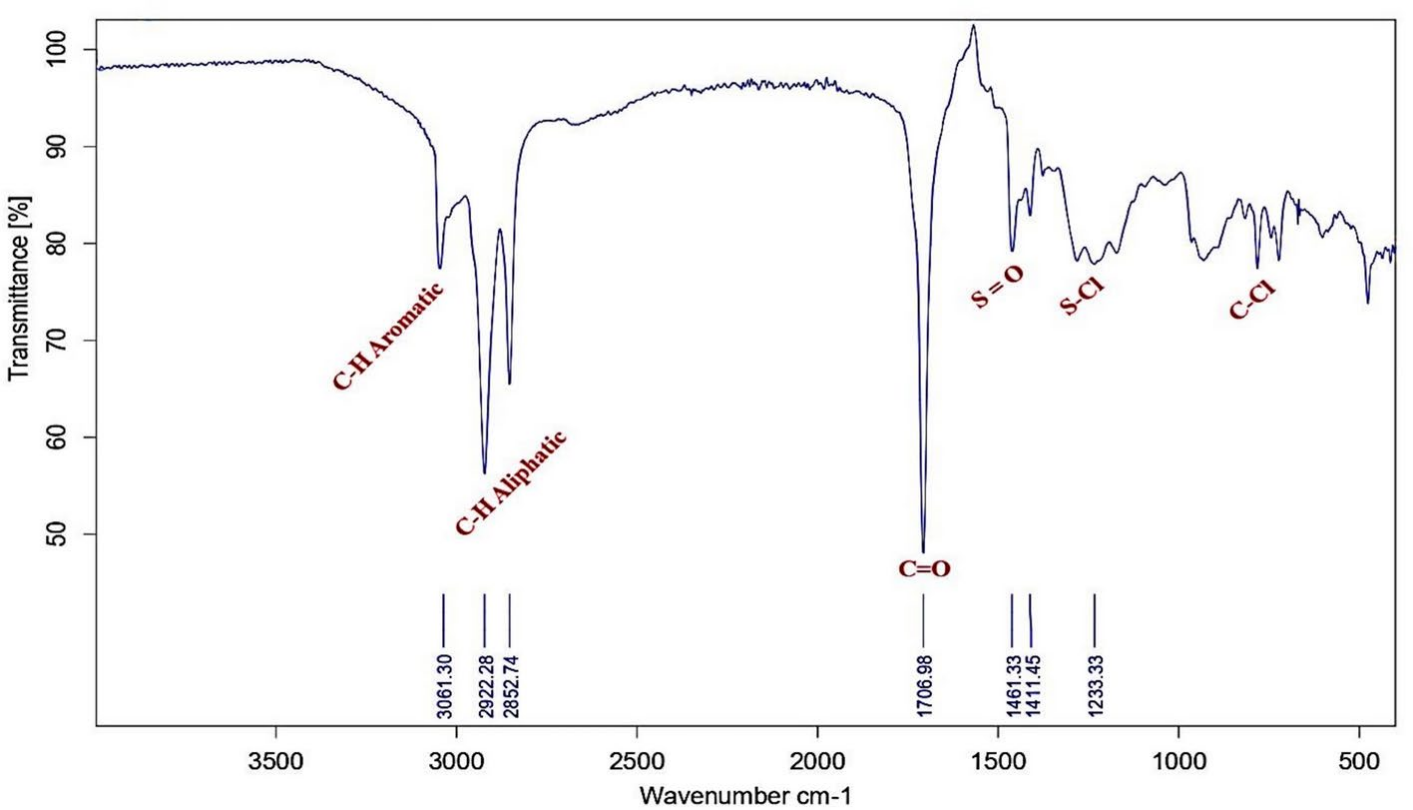
FT-IR of Chlorination Step of 10, NOAS.

The FTIR absorption bands analysis of the prepared bi-zwitterionic surfactants was recorded using a Thermo Fisher Scientific Spectrometer (400–4000 cm^-1^) with the horizontal axis representing the wave number in cm⁻^1^ and the vertical axis indicating the transmittance percentage. Figure 3. which represents the alkylation of naphthalene by oleic acid, shows abroad band at υ = 3392cm^-1^, indicating the presence of OH group. The aromatic C-H stretching peak was observed at υ = 3049 cm^-1^, while the aliphatic C-H bond appeared at υ = 2923 & 2852 cm^-1^. The absence of a peak at υ = 1640–1680 cm^-1^, associated with the C = C of oleic acid, indicates that the alkylation reaction was successfully conducted, resulting in the product **10, NOA**. Regarding to material **10, NOAS, **Fig. 4 shows a peak at υ = 600—700 cm^-1^ correspond to the C-S stretching bond indicating that the sulphonation occurred. The main feature of this peak demonstrated that the **10, NOAS** was also successfully prepared. In Fig. 5 for material **10, NOSC ,** the absence of the broad band related to OH groups and the appearance of a peak at υ = 800 cm^-1^ indicating the presence of the C–Cl stretching bond.

**Fig. 6:**
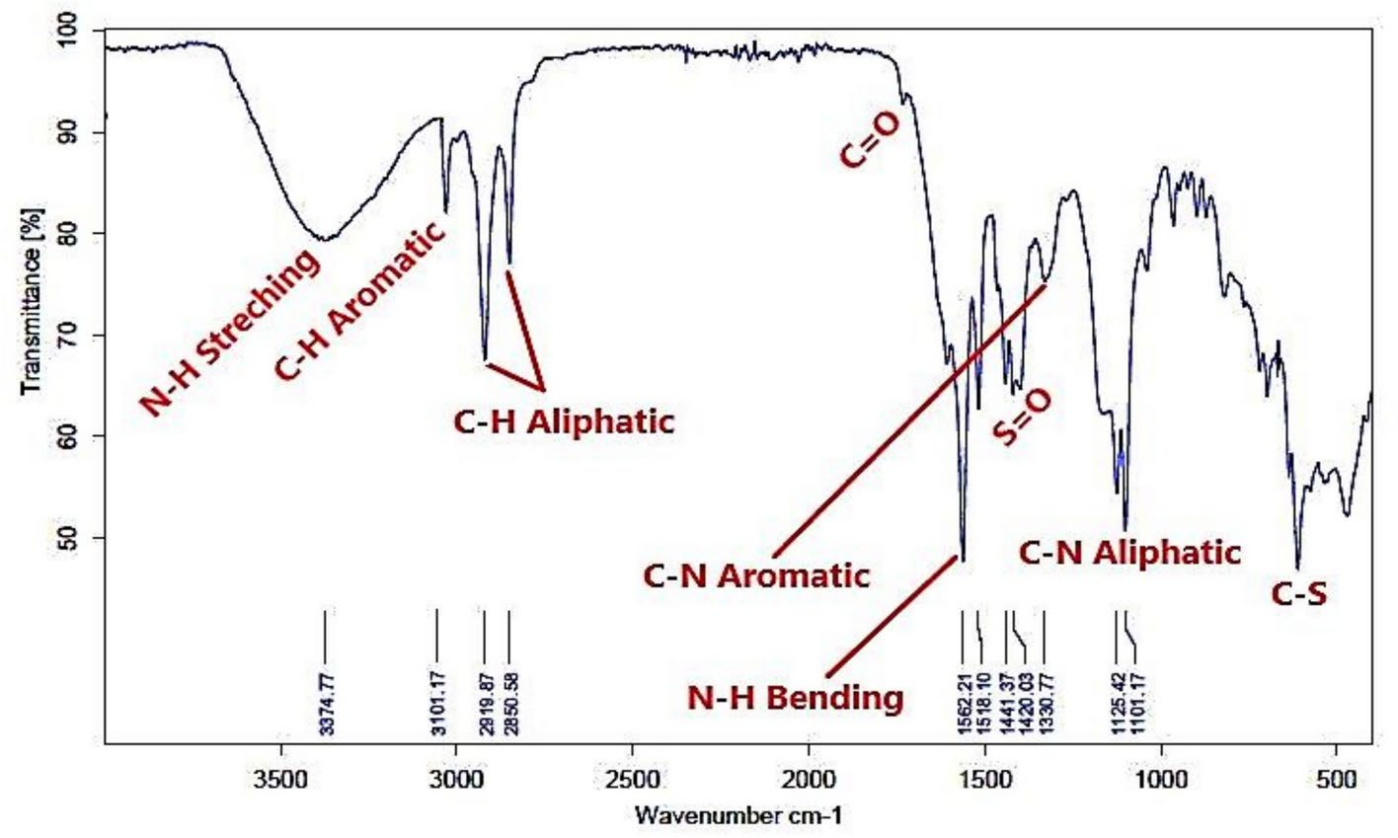
FT-IR of BPh.

**Fig. 7:**
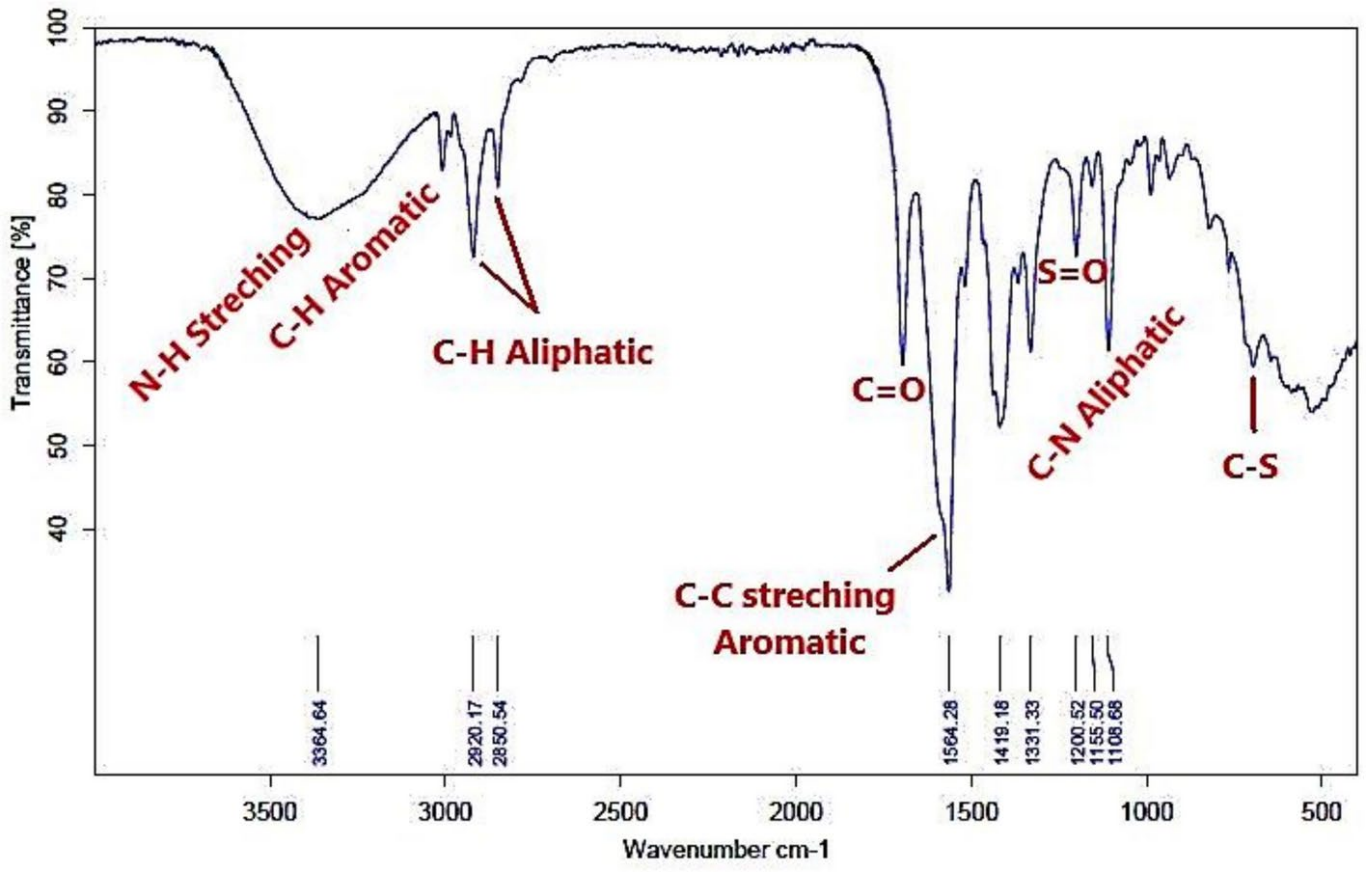
FT-IR of NPh.

Figures 6, 7 show the FT-IR of representative samples from the prepared bi-zwitterionic surfactants. There are N–H broad band ranged from υ = 3364 to 3431 cm^-1^ and C-N aliphatic from υ = 960 to 1165cm^-1^ . All these peaks demonstrate that BE, BP, BPh, NE, NP, and NPh were successfully synthesized.

#### Nuclear magnetic resonance (^1^H-NMR)

**Fig.8:**
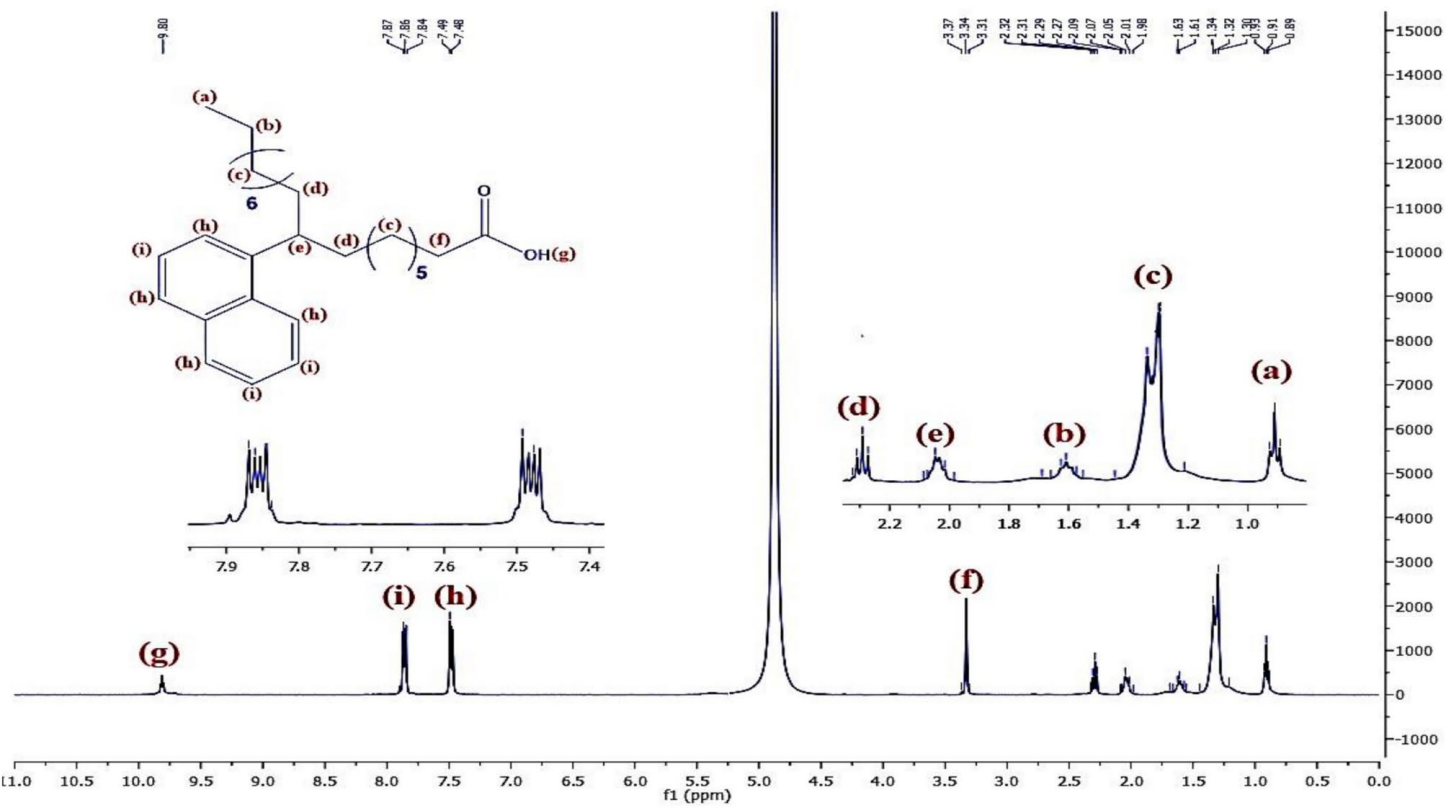
^1^H-NMR of Alkylated Naphthalene by Oleic Acid.

**Fig.9:**
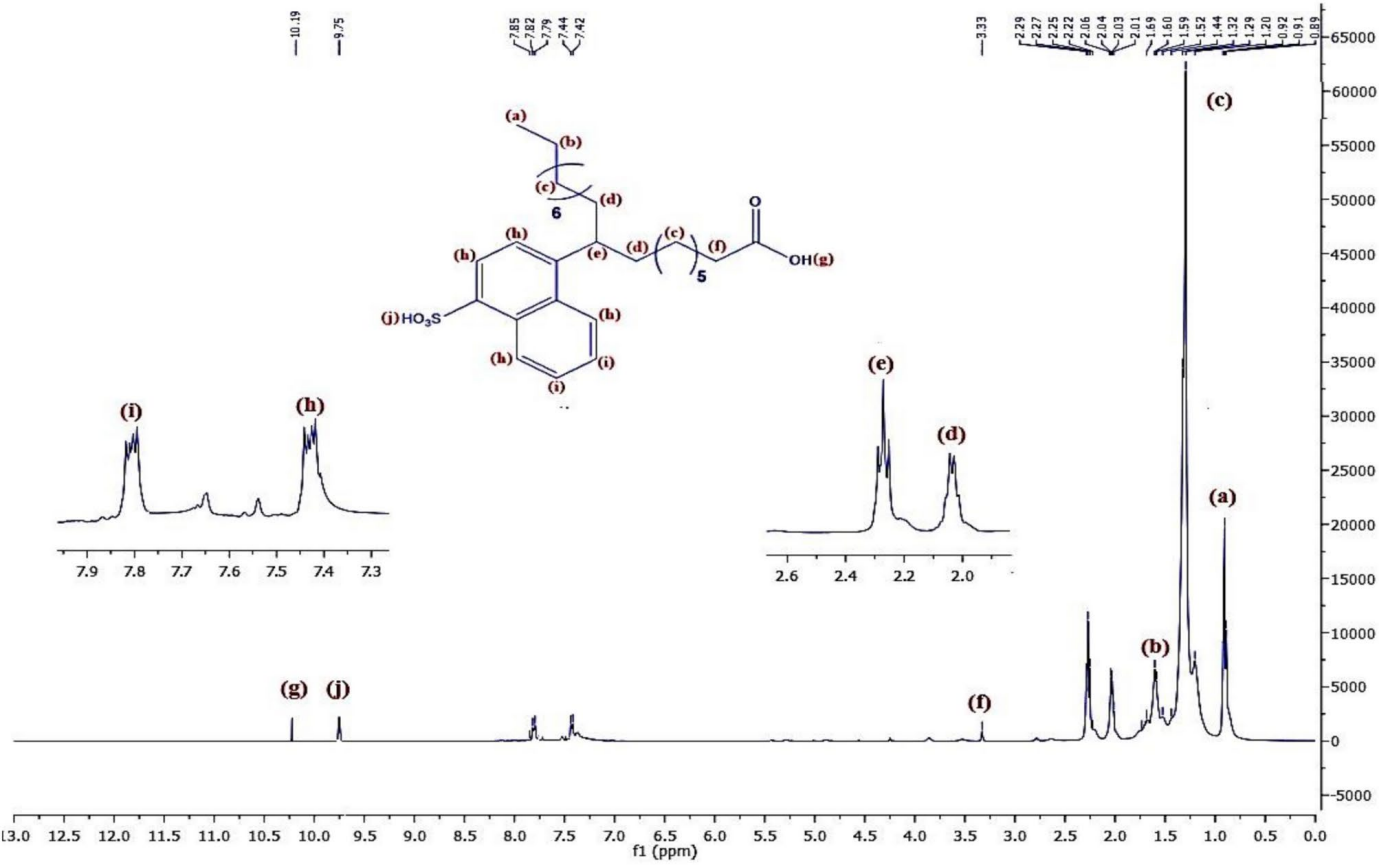
^1^H-NMR of Sulphonation Step of 10, NOA.

**Fig. 10:**
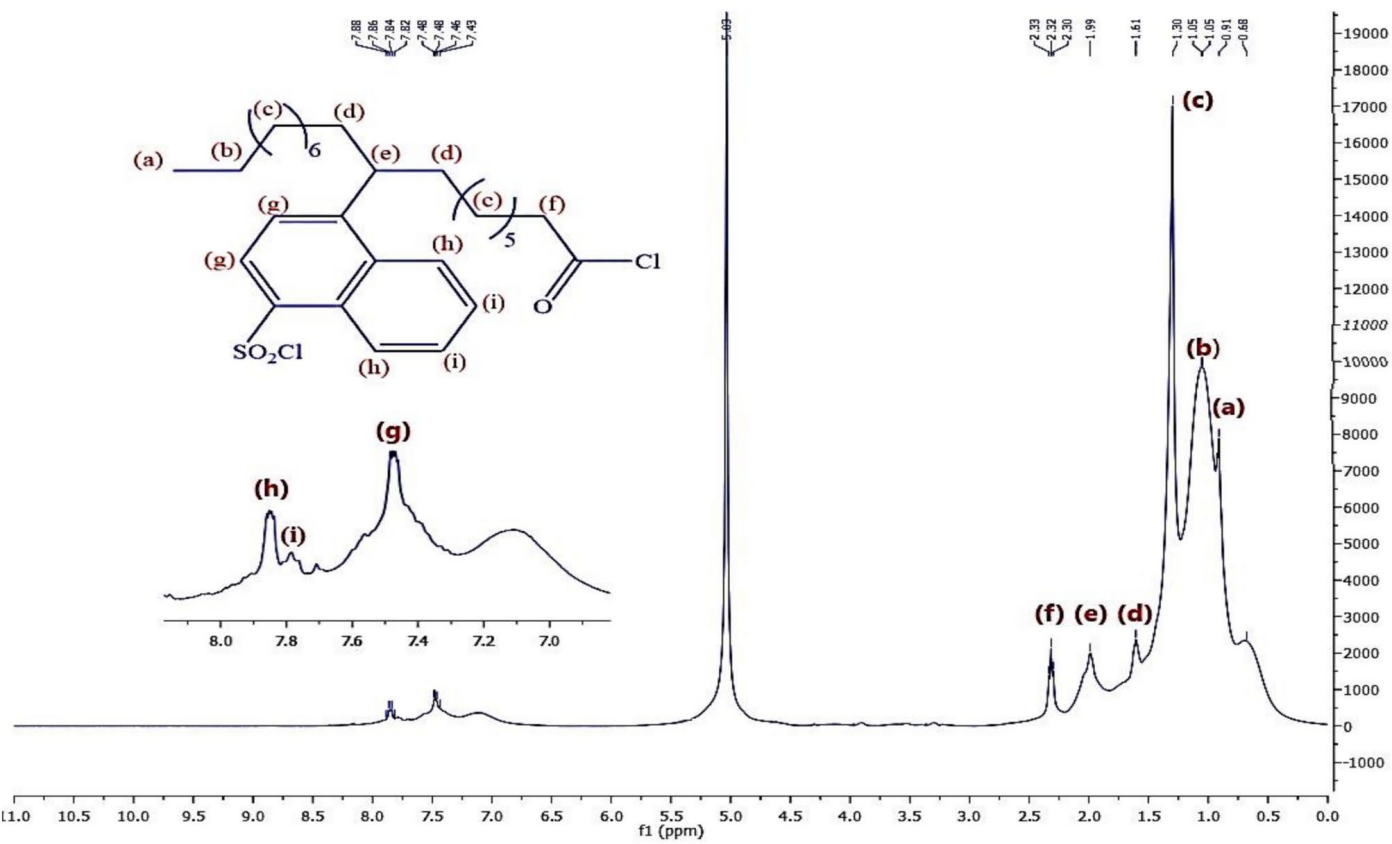
^1^H-NMR of Chlorination Step of 10, NOAS.

**Fig.11:**
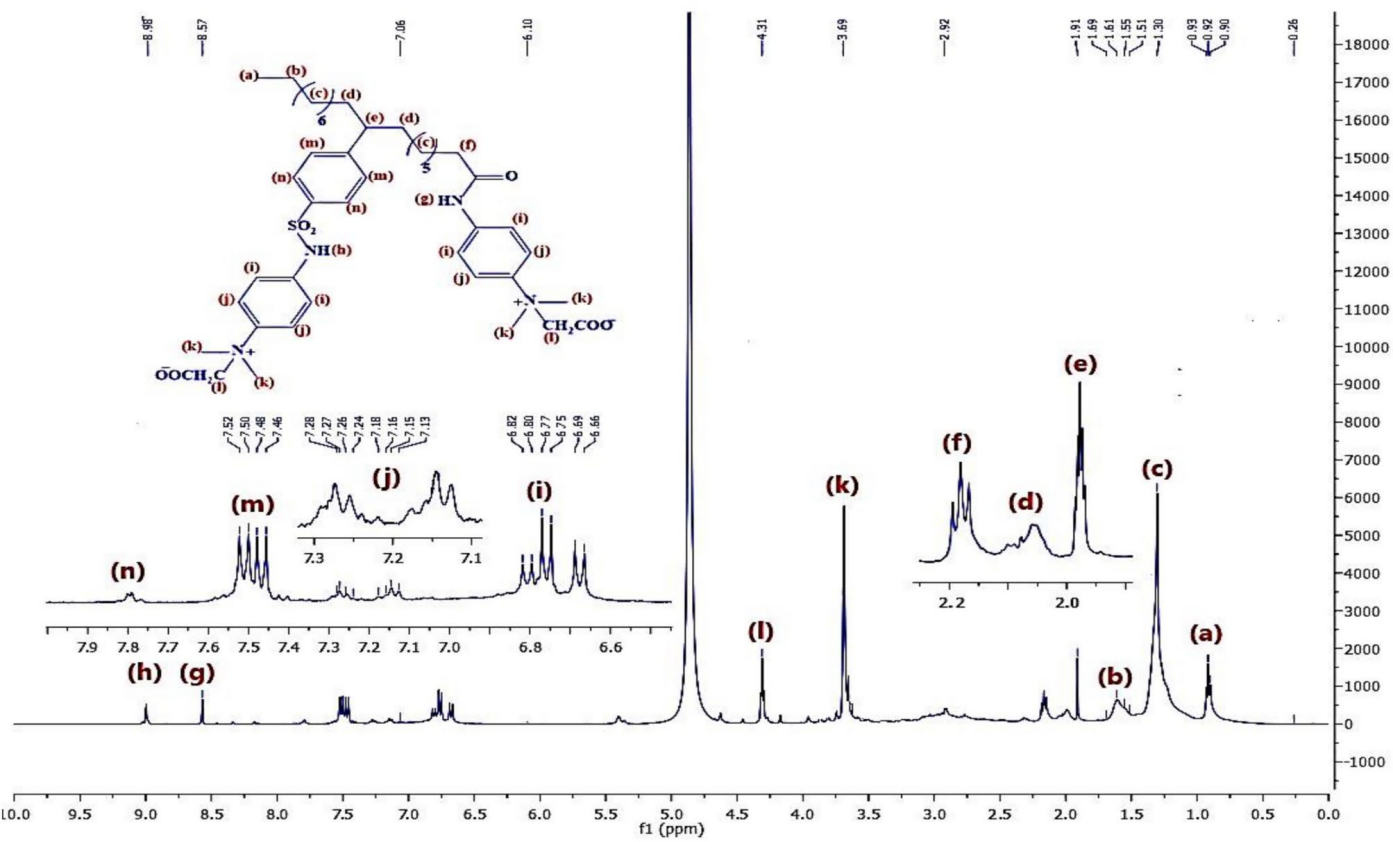
^1^H-NMR of BPh.

**Fig.12:**
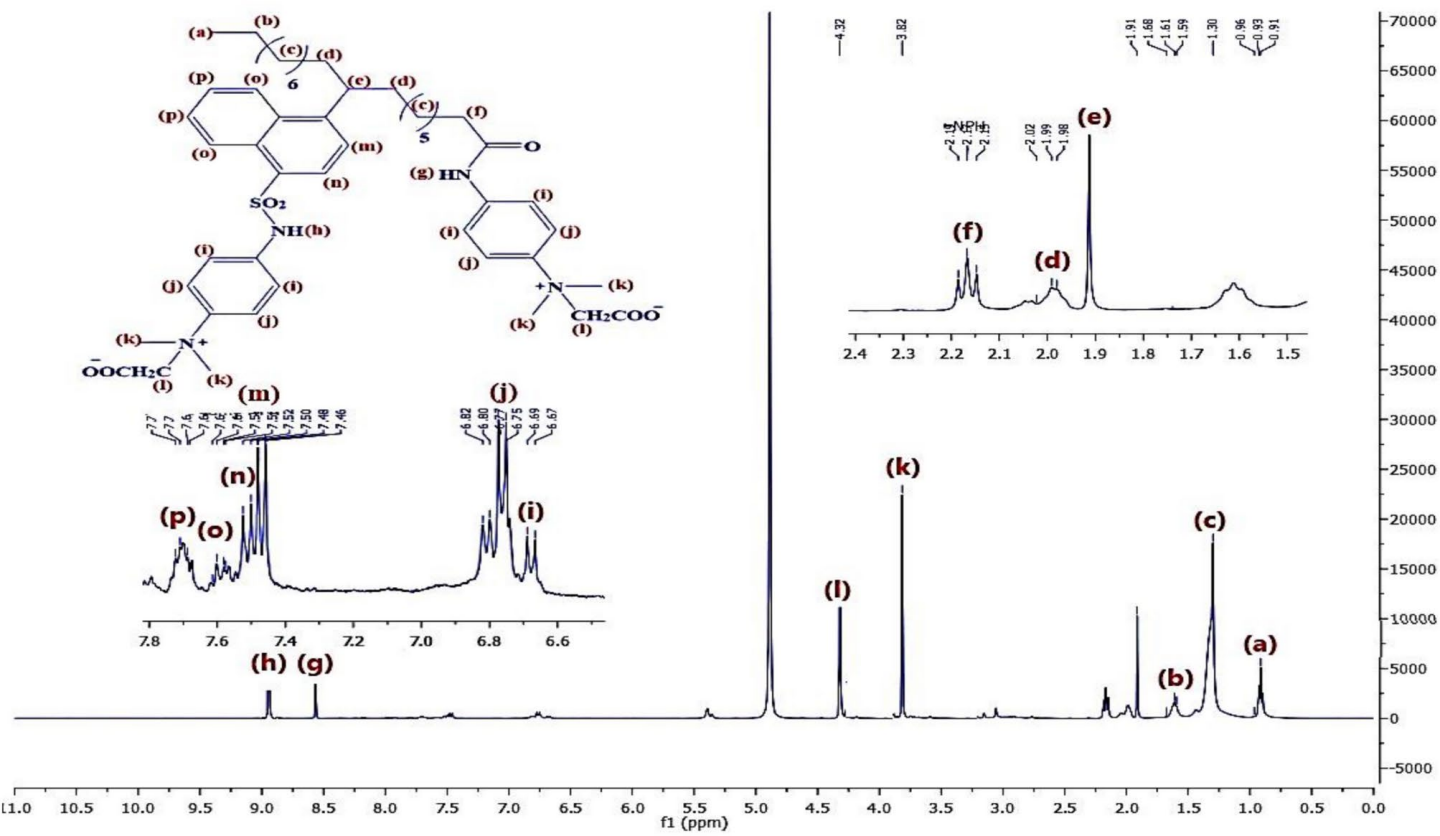
^1^H-NMR of NPh.

The ^1^H-NMR spectroscopy of the synthesized surfactants was conducted using a Bruker Avance 400 spectrometer (400 MHz) in D_2_O at ambient temperature, with tetramethylsilane (TMS) serving as a reference. The ^1^H-NMR chemical shifts of alkylation, sulphonation, and chlorination steps are presented as following:

Figure 8 represents the ^1^H-NMR of material **10, NOA,** which shows chemical shifts at δ = 0.91 ppm related to 3H^+^(a), δ = 1.6 ppm related to 2H^+^(b),δ = 1.32 ppm related to 2H^+^(c), δ = 2.3 ppm related to 2H^+^(d), δ = 2.03 ppm related to H^+^(e), δ = 3.34 ppm related to 2H^+^(f), δ = 9.7 ppm related to H^+^(g),δ = 7.5 ppm related to H^+^(h)& δ = 7.85 ppm related to H^+^(i). Regarding to material **10, NOAS, **Fig. 9 shows that there is a chemical shift appeared at δ = 9.75 ppm related to H^+^(j) which corresponding to the ^+^SO_3_H proton and this indicated to that sulphonation was occured. In Fig. 10 for **10, NOSC ,** the absence of the chemical shifts (j) and (g) as appeared in Fig. 9 of sulphonation indicated to successfully formation of -COCl and -SO_2_Cl groups. Figures 11, 12 show representative samples of the H^1^-NMR of the prepared bi-zwitterionic surfactants. According to Fig. 11, for surfactant **BPh**, there are four aromatic H^+^s that have chemical shifts at δ = 6.77 ppm related to 2H^+^ (i), δ = 7.18 ppm related to 2H^+^ (j), δ = 7.5 ppm related to 2H^+^(m) & δ = 7.8 ppm related to 2H^+^(n). In Fig. 12, the surfactant **NPh** exhibits chemical shifts analogous to BPh, but was characterized by additional shifts ranges δ = 7.55–7.8 ppm corresponding to 4H^+^ (o& p).

The FTIR and H^1^-NMR spectra confirmed the anticipated chemical structure of the bi-zwitterionic surfactants, demonstrating successful preparation.

### Surface active properties of the examined surfactants

**Fig.13:**
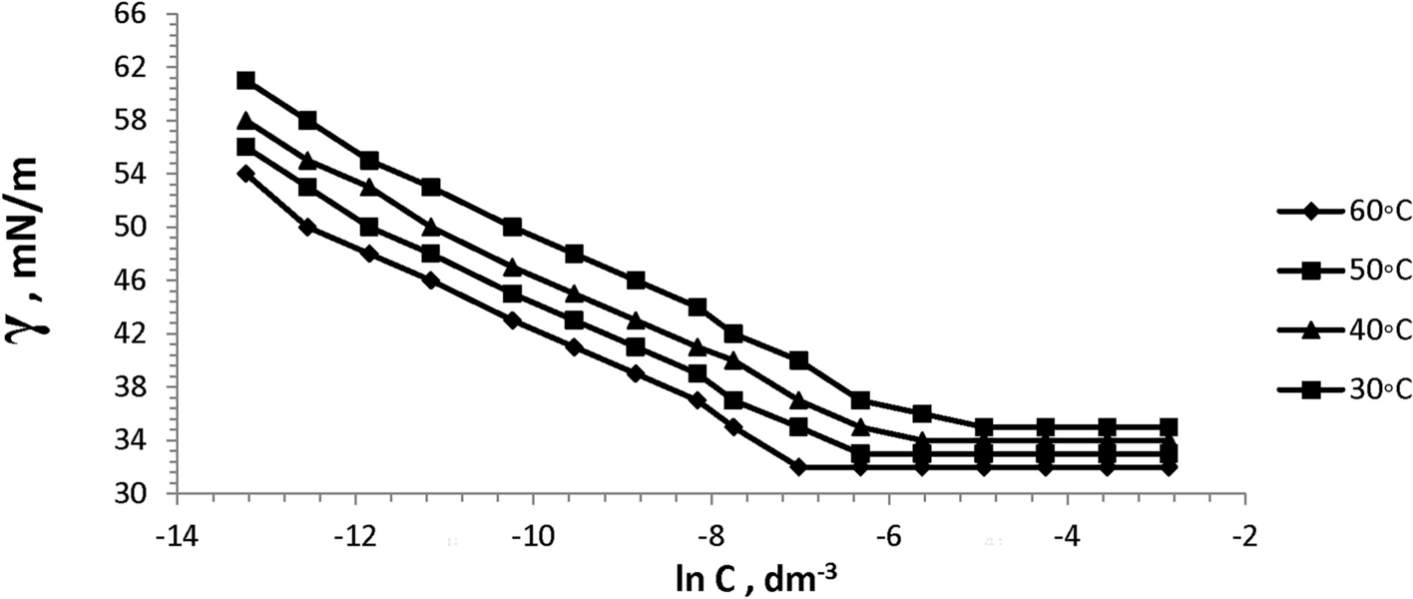
γ-ln C isotherm of BE at Different Concentrations and Temperatures.

**Fig.14:**
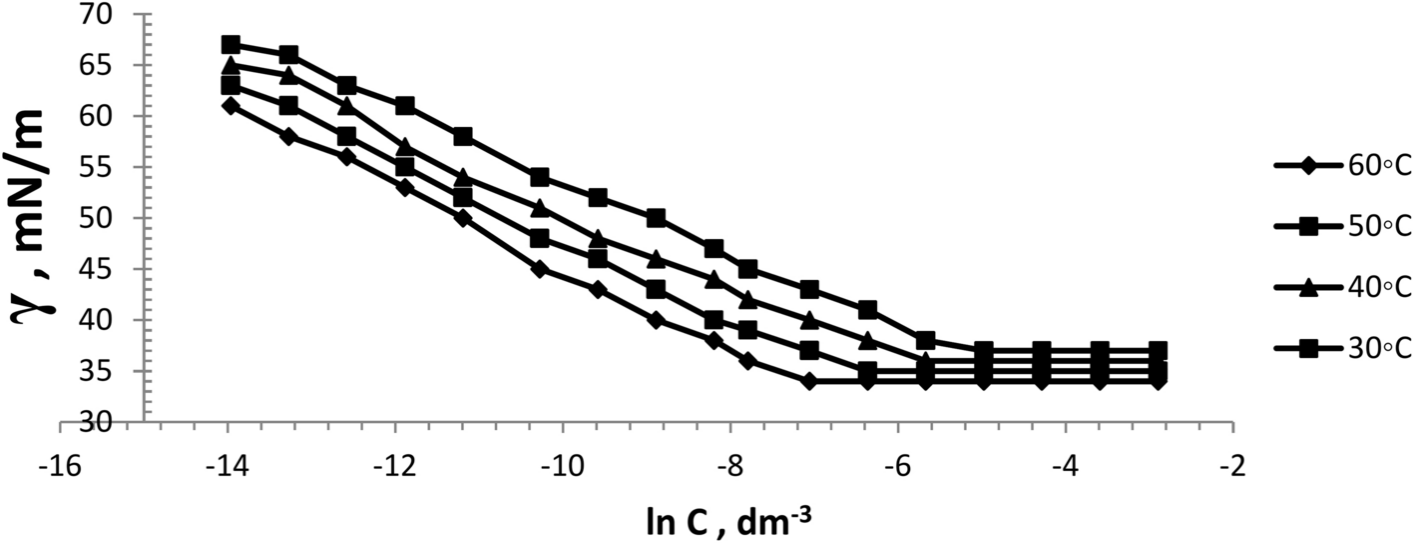
γ-ln C isotherm of BP at Different Concentrations and Temperatures.

**Fig.15:**
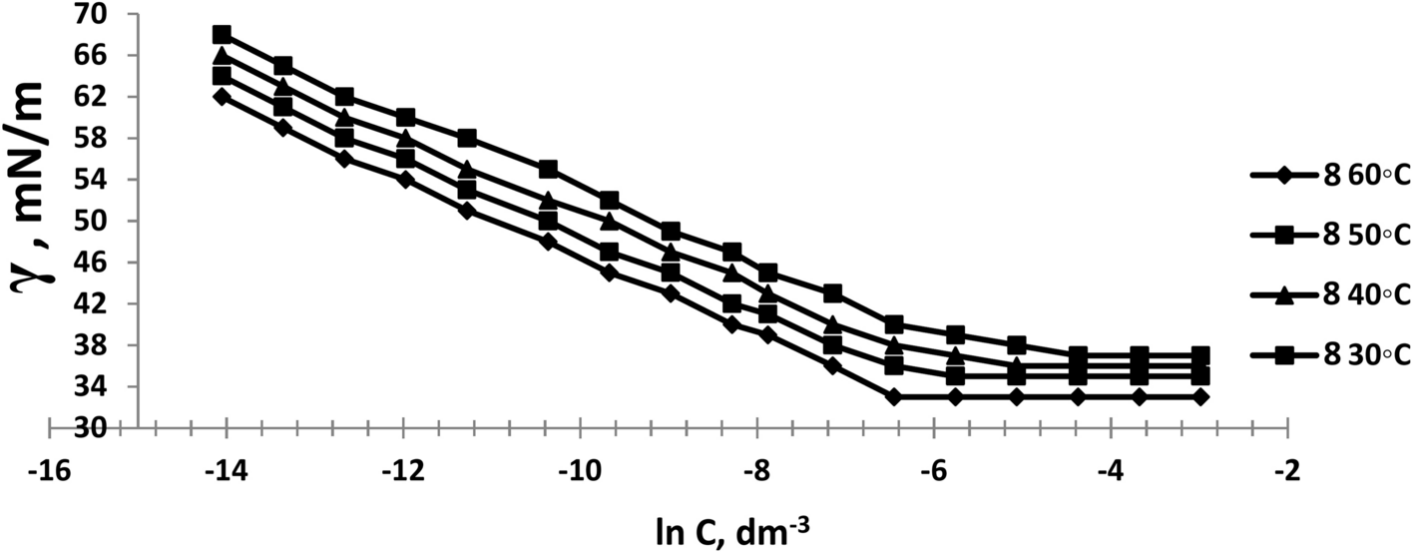
γ-ln C isotherm of BPh at Different Concentrations and Temperatures.

**Fig.16:**
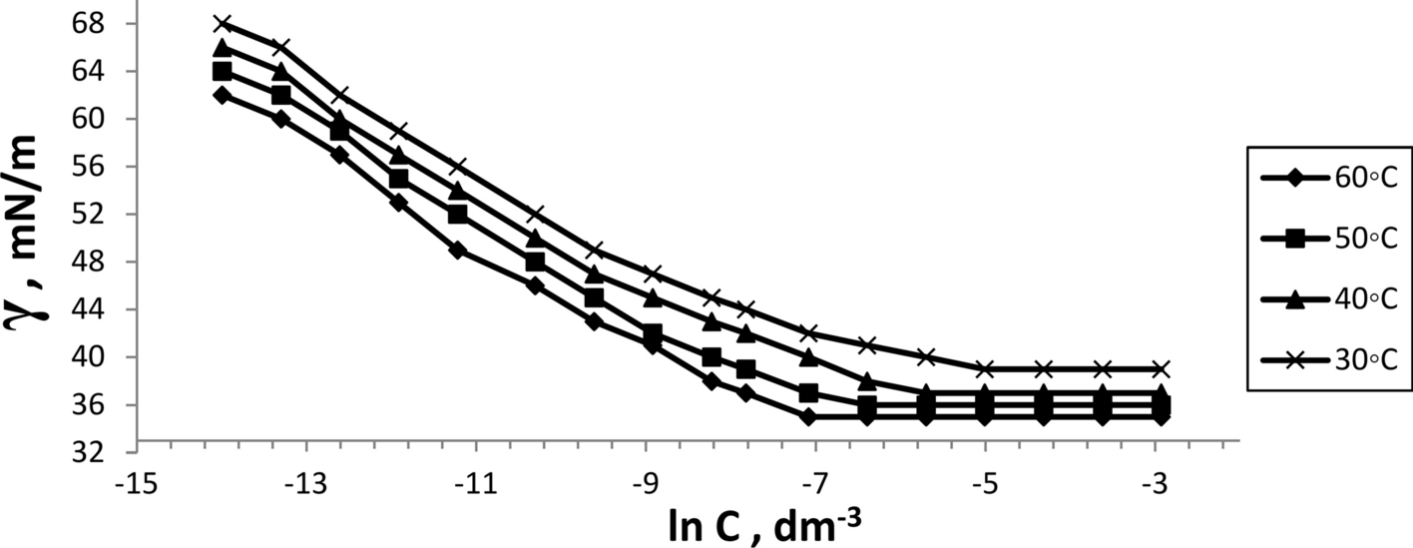
γ-ln C isotherm of NE at Different Concentrations and Temperatures.

**Fig.17:**
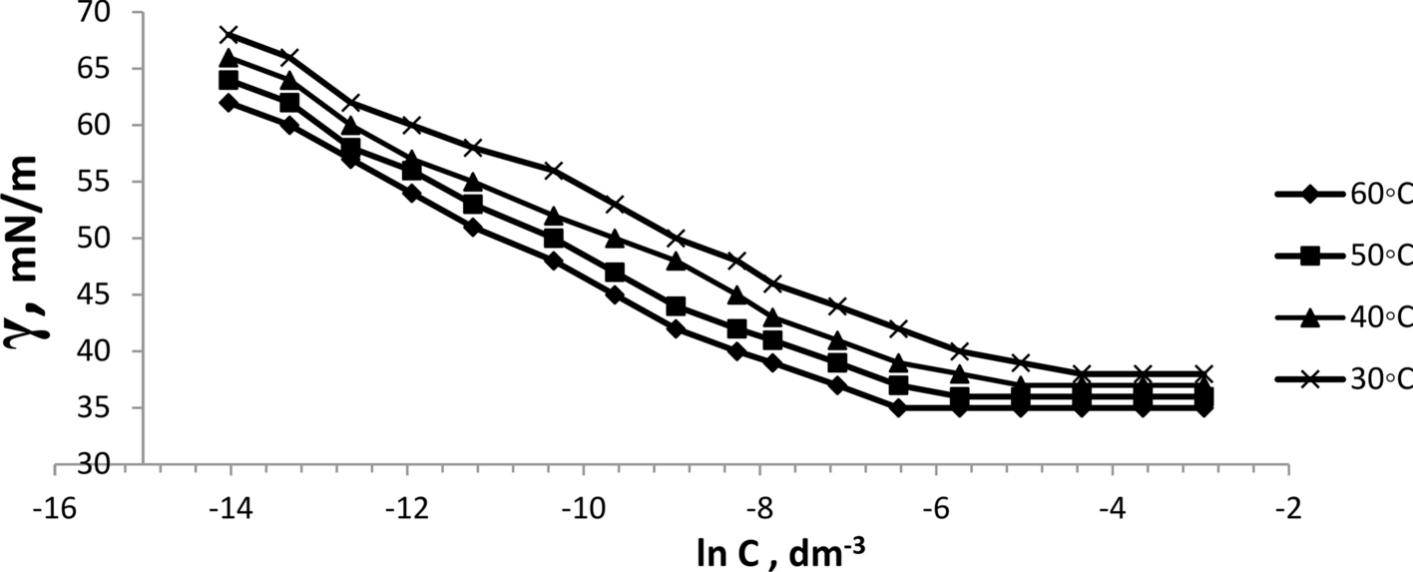
γ-ln C isotherm of NP at Different Concentrations and Temperatures.

**Fig.18:**
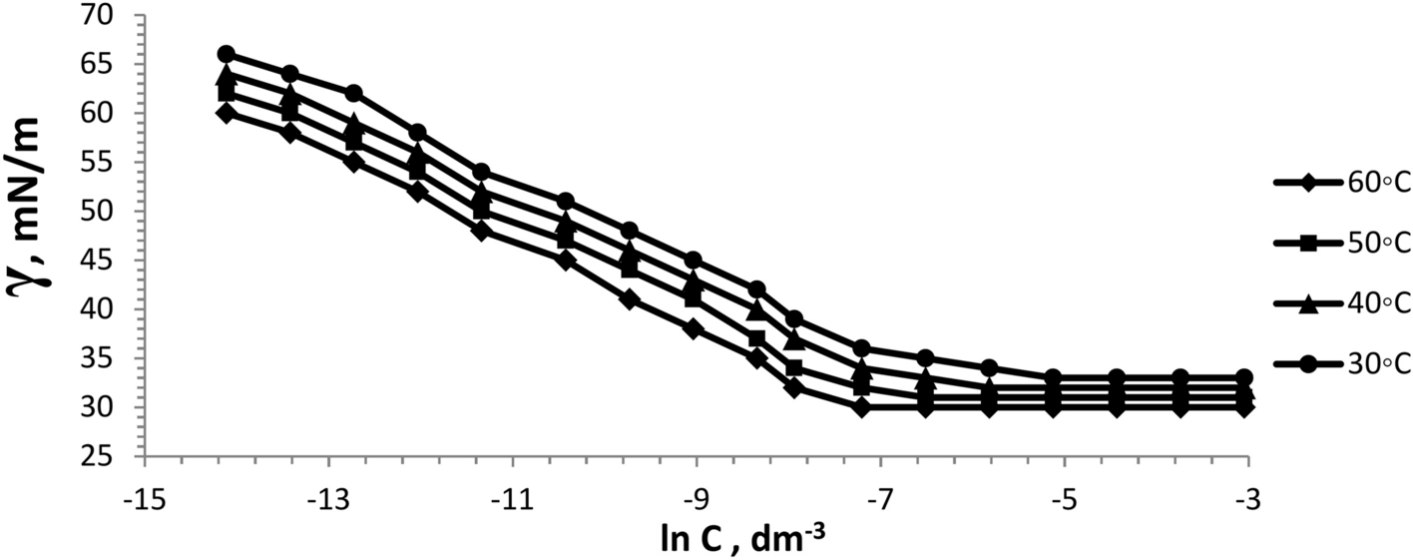
γ-ln C isotherm of NPh at Different Concentrations and Temperatures.

The surface tension of the prepared six bi-zwitterionic surfactants was measured at different concentrations and different temperatures of 30, 40, 50, and 60°C in formation water of TDS (2*10^5^ ppm). The surface tension against (-ln concentration), (dγ/dlnC), of the prepared surfactants was plotted as shown in Figs. 13, 14, 15, 16, 17, 18. Values of **CMC,** surface tension at CMC **(γ**_**CMC**_**), Pc**_**20**_**, and π**_**CMC**_ were obtained at the plot’s splitting point, and their values are displayed in Table 1.

**Table 1:** Surface active properties of the prepared surfactants.

Surfactant	Temp	Pc_20_	CMC, mol dm^-1^ (*10^–3^)	γ_cmc_ , (mNm^-1^)	π_cmc_ , (mNm^-1^)	A min, (A°^2^/molecule)	Γ_max_ , mol/cm^2^ (*10^–9^)
BE	30	4.430	5.46	35	34	14.882	1.116
	40	4.820	2.87	34	35	16.510	1.006
	50	5.103	1.29	33	36	17.865	0.929
	60	5.428	0.91	32	37	19.443	0.853
BP	30	3.822	4.74	37	32	13.608	1.22
	40	4.343	2.73	36	33	14.848	1.12
	50	4.603	1.50	35	34	16.116	1.03
	60	4.842	0.78	34	35	17.573	0.945
BPh	30	3.952	6.08	37	32	13.834	1.20
	40	4.169	3.18	36	33	14.739	1.126
	50	4.473	2.04	35	34	15.733	1.055
	60	4.755	1.66	33	36	16.160	1.027
NE	30	4.234	4.51	39	30	15.107	1.1
	40	4.430	2.07	37	32	15.468	1.07
	50	4.625	1.10	36	33	16.414	1.01
	60	4.907	0.61	35	34	17.758	0.935
NP	30	3.865	8.39	38	31	14.278	1.16
	40	4.169	4.08	37	32	15.334	1.08
	50	4.516	2.02	36	33	16.312	1.02
	60	4.733	1.34	35	34	17.353	0.957
NPh	30	4.408	3.69	33	36	12.484	1.33
	40	4.625	1.83	32	37	13.350	1.24
	50	4.916	1.00	31	38	14.225	1.17
	60	5.059	0.61	30	39	15.277	1.09

The study examined the adsorption effectiveness of synthesized surfactants at the air/water interface by calculating surface-active properties, including the minimum surface area per molecule (A_min_), maximum surface excess concentration (Γ_max_), and thermodynamic parameters like Gibbs free energy of micellization (ΔG_mic_) and Gibbs free energy of adsorption (ΔG_ads_). The surface active properties were measured from the following equations:

The maximum surface excess concentration (Γ_max_) can be calculated from Eq. 1:

1 $$\Gamma_{{{\text{max}}}} \left( {mol/cm^{2} } \right) \, = \, - {1}0^{{ - {7}}} \left[ {{1}/{\text{RT}}} \right] \, \left[ {{\text{d}}\gamma /{\text{ dlnC}}} \right]$$

where T is the absolute temperature in kelvin = (T °C + 273) K; C is the prepared surfactant concentration (mol/l), and R is the gas constant = 8.31 J mol^-1^ K^-1^. The minimum surface area per molecule can be calculated from Eq. 2:

2 $${\text{A}}_{{{\text{min}}}} \left( {A^{ \circ 2} /molecule} \right) \, = { 1}0^{{{16}}} / \, \left[ {\Gamma_{{{\text{max}}}} .{\text{N}}_{{\text{A}}} } \right]$$

where N_A_ is the Avogadro’s number = 6.023 × 10^23^ molecule/mol.

The value of (dγ/dlnC) is derived from the graph slope of surface tension against ln [concentration] below the CMC.

The adsorption efficiency (Pc_20_) can be obtained by the negative logarithm of the surfactant concentration, which decreases the surface tension of the pure solvent by 20 mN/m. The adsorption efficiency is calculated by Eq. 3:

3 $${\text{Pc}}_{{{2}0}} = \, - {\text{ log C}}_{{{2}0}}$$

where C_20_ is the concentration of surfactant necessary to decrease the surface tension of formation water by 20 mN/m, signifying that C_20_ is the minimum concentration indicating adsorption saturation at the surface. C_20_ indicates the adsorption effectiveness of surfactant molecules at the air–water interface. Therefore, increased values of Pc_20_ correlate with great adsorption of surfactant molecules^31^.

The surface pressure at the CMC (π_cmc_) is calculated from Eq. 4:

4 $$\pi_{{{\text{cmc}}}} = \gamma_{{\text{o}}} {-}\gamma_{{{\text{cmc}}}}$$

where **γ**_**o**_ is the surface tension of the used water, and **γ**_**cmc**_ is the surface tension of the surfactants at the CMC.

Figures 13, 14, 15, 16, 17, 18 demonstrate the decrease in surface tension of surfactant solutions at various concentrations. The graph shows that an increase in surfactant concentration leads to a decrease in surface tension values until it reaches the CMC. At 60 °C, the surfactant NPh had the lowest surface tension of 30mN/m at the concentration of 625 mol.dm^-3^ (ppm). Surface tension decreases due to increasing the adsorption of surfactant molecules at the air–water interface ^32^. Surfactant particles are adsorbed at the air–water interface at which the hydrophilic head group faces the water phase, and the hydrophobic carbon chain faces the air phase. The surfactant particles adsorption at the interface increases as surfactant concentration increases. After reaching the critical micelle concentration (CMC), surfactant adsorption at the surface does not increase further^33^, indicating that higher surfactant concentrations do not lead to a reduction in surface tension.

Table 1 indicates that the synthetic surfactant **NPh** exhibits the highest surface excess concentration (Γ_max_) and the lowest area per molecule (A_min_) across all temperatures among the surfactants analyzed. These surfactants exhibit high (Γ_max_) values due to their large hydrophilic head groups^34^. The reduction in area value per molecule may result from the crowding of the hydrophilic heads of surfactants at the interface, leading to compaction, alongside the overlapping of hydrophobic chains^35^. Increasing temperature results in a rise in A_min_/molecule and a decrease in Γ_max_, which is a significant characteristic in oil extraction due to the increase of surfactant molecules at the interface. This may be lead to more oil solubilization, yielding favorable outcomes for the oil producion. It also may facilitate the formation of thermodynamically stable micro-emulsions and increasing the oil migration and the recovery factor (RF).

### Thermodynamic Characteristics of Micellization and Adsorption:

Thermodynamic parameters like ΔG_mic_ and ΔG_ads_ values have been obtained using the Gibbs free energy Eqs. 5& 6 and listed in Table 2.

**Table 2:** Thermodynamic Parameters of Adsorption and Micellization of the Prepared Surfactants:

Surfactant	Temp	∆G_mic_, kj/mol	∆S_mic_	∆H_mic_	∆G_ads_, kj/mol	∆S_ads_	∆H_ads_	Structure Effect
BE	30	-13.11	0.2139	51.85	-16.16	0.2563	61.67	3.04
	40	-15.21		51.73	-18.69		61.55	3.47
	50	-17.84		51.69	-21.72		61.51	3.87
	60	-19.37		51.24	-23.70		61.08	4.33
BP	30	-13.47	0.2104	50.52	-16.09	0.2463	66.54	2.62
	40	-15.34		50.50	-18.29		66.53	2.92
	50	-17.44		50.32	-20.74		66.41	3.29
	60	-19.78		50.29	-23.48		66.38	3.70
BPh	30	-12.84	0.1626	35.95	-15.50	0.1906	42.28	2.66
	40	-14.95		35.92	-17.88		42.27	2.92
	50	-16.61		36.45	-19.83		41.80	3.22
	60	-17.71		36.44	-21.21		41.75	3.50
NE	30	-13.59	0.2276	55.37	-16.32	0.2576	61.73	2.72
	40	-16.07		55.35	-19.05		61.70	2.98
	50	-18.27		55.24	-21.54		61.67	3.26
	60	-20.44		55.17	-24.08		61.58	3.63
NP	30	-12.03	0.2110	51.98	-14.70	0.2405	58.25	2.66
	40	-14.30		51.90	-17.26		58.17	2.95
	50	-16.64		51.74	-19.88		58.02	3.24
	60	-18.29		51.52	-21.84		57.80	3.55
NPh	30	-14.10	0.2126	50.33	-16.80	0.2419	56.48	2.70
	40	-16.38		50.30	-19.36		56.45	2.97
	50	-18.52		50.17	-21.77		56.35	3.25
	60	-20.47		50.16	-24.06		56.31	3.58

Gibbs free energy for micellization:

5 $$\Delta {\text{G}}_{{{\text{mic}}}} = {\text{ R T ln CMC}}$$

Gibbs free energy for adsorption:

6 $$\Delta {\text{G}}_{{{\text{ads}}}} = \, \Delta {\text{G}}_{{{\text{mic}}}} - \, [{6}.0{23 } \times \pi_{{{\text{cmc}}}} \times {\text{ A}}_{{{\text{min}}}} ]$$

Negative ΔG_mic_ values indicate the micelle creation in the solution’s bulk phase, indicating that micellization is a spontaneous association-dissociation process at the interface. Negative ΔG_mic_ values also increase solvent-free energy, causing surfactant molecules to tend to surface and interface adsorption before micelle formation. The negative ΔG_ads_ values imply that surfactant molecules are adsorbed at the interface spontaneously. The increased negativity of ΔG_ads_ is attributed to increasing the curvature of the aqueous surface, which results in greater availability of vacancy sites for adsorption. With rising temperatures, the amount of surfactant molecules adsorbed at the surface or interface increases, as shown in Table 2. The results suggest that these surfactants are likely to produce stable emulsions at the critical micelle concentration (CMC). The structure effect can be obtained from the following equation:

7 $${\text{Strucural Effect }} = \, \Delta {\text{G}}_{{{\text{ads}}}} - \, \Delta {\text{G}}_{{{\text{mic}}}}$$

The negative values of ΔG_mic_ indicate that the micellization process is spontaneous process, while ΔG_ads_ also exhibits negative values. This indicates that both adsorption and micellization are co-occurring. The positive values of the structural effect obtained from Eq. 7 indicate that the magnitude of Gibbs’s energy of adsorption at the air–water interface is more exuberant than the formation of micelles in the entire solution mass^36,37^.

This theory indicates that the adsorption process initially occurs on the surface until monolayer adsorption is complete, after which the molecules aggregate toward the bulk of the solution to form micelles^38^. This hypothesis is corroborated by the more negative values of ΔG_ads_ compairing to ΔG_mic_.

The significant positive value of the structural effect may indicate effective interfacial interaction with the surrounding media^39,40^. The **BE** surfactant demonstrated the highest structural effect among all zwitterionic surfactants. The elevated values of the structural effect may be attributed to the presence of both cationic and anionic moieties within the molecule.

The standard change of entropy ΔS values of adsorption and micellization can be obtained separately using the slope (*dΔG*_*ads*_*/dT*) and (*dΔG*_*mic*_*/dT*), respectively, at different temperatures.

**Fig.19:**
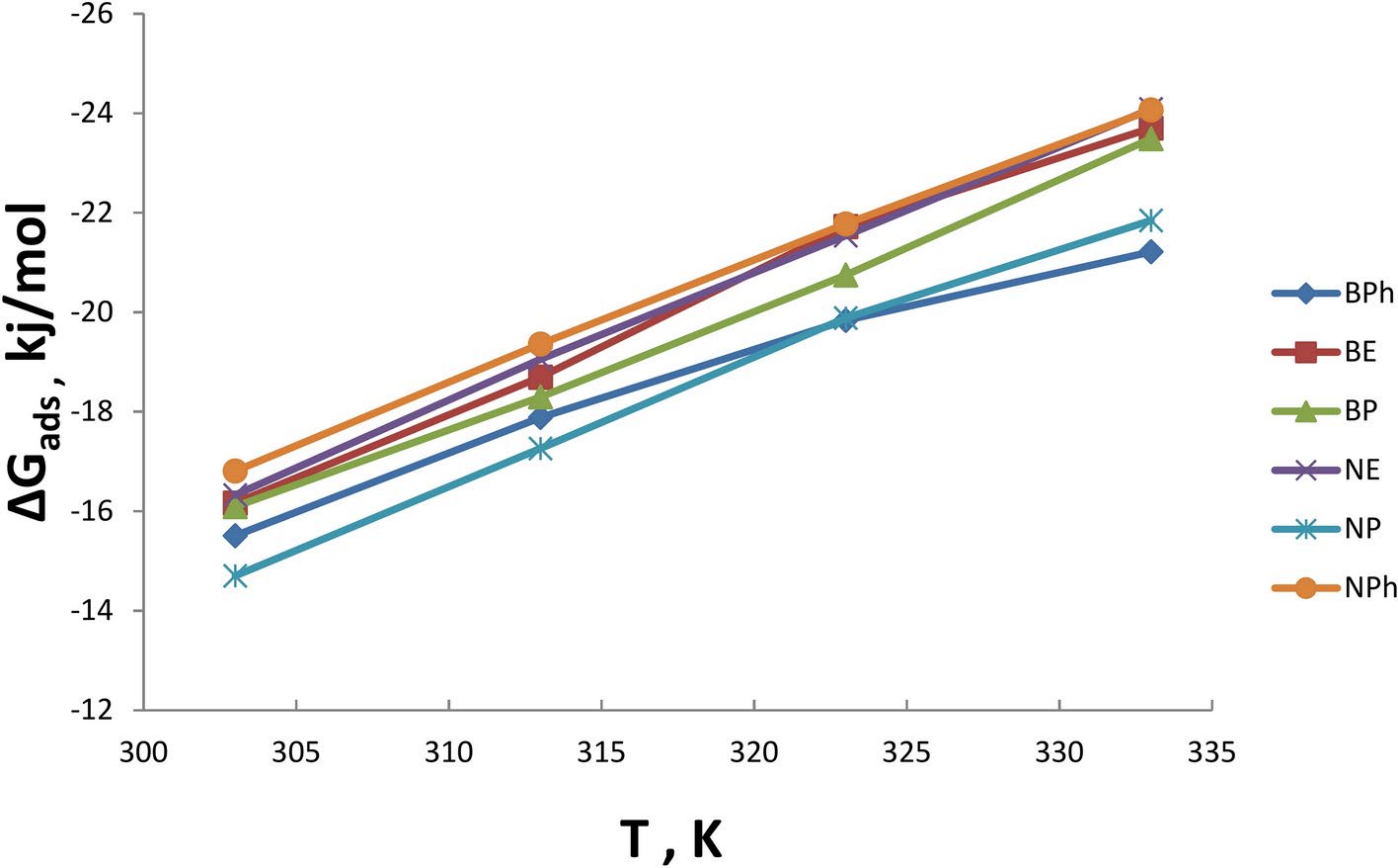
Entropy of Adsorption of the Prepared Surfactants at Different Temperatures.

**Fig.20:**
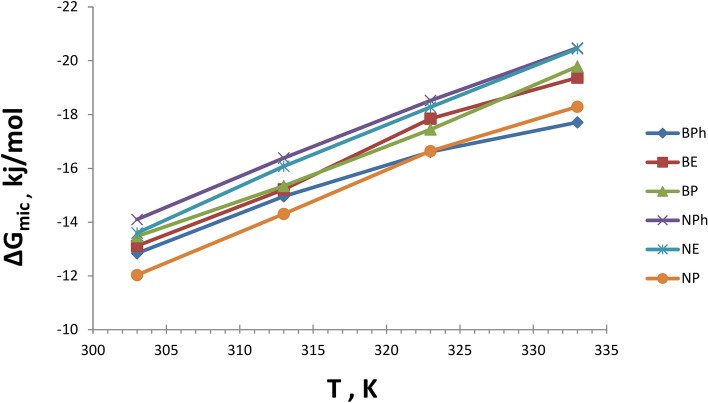
Entropy of Micellization of the Prepared Surfactants at Different Temperatures.

The negative values of ΔG_mic_ and ΔG_ads_ are primarily attributable to the high positive values of ΔS_mic_ and ΔS_ads_. The entropy values for micellization and adsorption can be derived from the slope of the plot of (d∆G/dT), as shown in Figs. 19, 20. ΔH is often positive, and even if negative, it is significantly lower than TΔS_mic_ or TΔS_ads_. ΔH can be calculated from Eq. 8:

8 $$\Delta {\text{G}}_{{\text{ads or mic}}} = \, \Delta {\text{H}}_{{\text{ads or mic}}} + {\text{ T}}\Delta {\text{S}}_{{\text{ads or mic}}}$$

As a result, the micellization and adsorption processes are primarily governed by the gain of entropy associated with them. The surfactant alkyl group’s tendency to migrate from the solvent media to facilitate monolayer adsorption, followed by its return to form micelles, serves as the primary driving force. The significant entropy associated with micellization and adsorption in aqueous media can be explained as follows:

1. The alkyl chains are extracted from the aqueous medium and incorporated into the micelle, increasing the system’s entropy. This phenomenon is attributed to the arrangement of water molecules surrounding the alkyl chains, influenced by "Vander Wall Forces"^41,42^.
2. Increasing the freedom of the hydrophobicity of the alkyl chain in the non-polar interior of the micelle compared to the aqueous environment^43,44^. In an aqueous medium, a slight decrease in ΔH_mic_ and ΔH_ads_, occured with rising temperature, resulting in a more negative ΔG_mic_ and ΔG_ads_ by approximately 2.5 kJ per 10°C, as illustrated in Table 2.

Increasing the negativity of ΔG_mic_ and ΔG_ads_ values with temperature leads to modifying the degree of non-polarity of the internal structure of the micelle and also modifies the polarity of the hydrophilic head. Consequently, water penetrates the micelle, particularly in the first carbon atoms adjacent to the hydrophilic head^41,45^.

### Interfacial tension of the prepared surfactants

When assessing the efficiency of surfactants for enhanced oil recovery process, interfacial tension emerges as a critical parameter. The ability to enhance the oil recovery improves as IFT values decrease. The IFT results are presented in Table 3. The IFT at CMC was assessed in formation water with a TDS of 2*10^5^ ppm at temperatures of 50 and 70°C. The results indicate that the interfacial tension (IFT) decreases with increasing temperature. Surfactants with interfacial tension (IFT) values ranging from 10^–1^ to 10^–3^ mNm^-1^ are expected to be effective oil production in different petroleum fields . The analysis of the IFT data in Table 3 reveals that NE and NPh demonstrate the lowest IFT values at 50 °C.

**Table 3:** Interfacial tension of the examined surfactants:

Surfactant	IFT, mNm^-1^	
	50°C	70°C
BP	4*10^–1^	8*10^–2^
BE	2*10^–1^	6*10^–2^
BPh	3*10^–1^	7*10^–2^
NE	2*10^–2^	> 10^–2^
NP	4*10^–2^	> 10^–2^
NPh	3*10^–2^	> 10^–2^

Increasing the temperature from 50 to 70°C resulted in a marginal reduction in IFT values. This phenomenon may result from an increase in the free energy of the surfactant, facilitating the adsorption of surfactant molecules at the interface, thereby promoting oil solubilization and micro-emulsion formation, which may be lead to increase the oil production in different petroleum fields^46^.

## Summary of key findings

It is important to mention that, the surfactants which containing aromatic nuclus in the molecules have been commonly used in oil recovery^47^. This is due to that the existence of benzene ring in the head group would favor the surface tension reduction and has a great effect on the interfacial properties^48^ thus, the bio-based zwitterionic surfactants could be considered potent alternatives in oil recovery. From this point of view, in this study we added benzene rings for the first studied group of the prepared surfactants and naphthalene rings for the second group.

The mechanism of the prepared surfactants for reducing oil–water interfacial tension described the adsorbtion of molecules on the oil–water interface and form a self-assembled monolayer. This enhances the interfacial interactions including the surfactant hydrophilic head group–water interaction and surfactant hydrophopic chain–oil interaction. The interfacial properties are dependent on the surfactant molecular structure, especially the type of hydrophilic head group and the hydrophobic chain configuration.

In this work, the BE surfactant which has one phenylene ring and two ethylenediamine sites decreased the interfacial tension to 2*10^–1^ mNm^-1^ at 50°C and the BP surfactant which has one phenylene ring and two propylenediamine sites decreased the interfacial tension to 4*10^–1^ mNm^-1^ at 50°C , while the BPh surfactant has three phenylene rings and this surfactant decrease the interfacial tension to 3*10^–1^ mNm^-1^ at 50°C. On the other side, the NE surfactant has one naphthalene unit and two ethylenediamine sites leading to decrease the interfacial tension to 2*10^–2^ mNm^-1^ at 50°C, NP surfactant which has one naphthalene unit and two propylenediamine sites decreased the interfacial tension to 4*10^–2^ mNm^-1^ at 50°C and the BPh surfactant has one naphthalene unit and two phenylene rings and this surfactant decreased the interfacial tension to 3*10^–2^ mNm^-1^ at 50°C. From these data we can concluded that, the efficiency of the prepared surfactants to reduce the surface tension and interfacial tension can be ranked as following **NE < NPh < NP < BE < BPh < BP**. All these data may be due to their large hydrophilic head groups at which the crowding of the hydrophilic heads of these surfactants at the interface leads to compaction alongside the overlapping of the hydrophobic chains. This may lead to more oil solubilization, yielding favorable outcomes for the oil producion. It also may facilitate the formation of thermodynamically stable emulsions and increasing the recovery factor (RF).

## Conclusion

In this study, six bi-zwitterionic surfactants were synthesized from oleic acid, by alkylation ,sulphonation, chlorination, amidation and finally quaternization of the two amide sites on the same molecule. The chemical structure of these surfactants was confirmed by the FTIR and H^1^-NMR spectroscopy. The physicochemical properties were studied based on surface tension measurement. Their critical micelle concentration (CMC) was notably low at range about 625 ppm (mol.dm^-3^) and 60 °C, which rendering them for application in petroleum industry.

The surface activity and thermodynamic properties of the synthesized surfactants were investigated on the bases of surface tension measurments at different temperatures . The data indicated that, the all surfactants reduce γ_cmc_ values, resulting in improving the surface activity and thermodynamic properties.

The study also revealed that, the values of ∆G_ads_ are more negative than those of ∆G_mic_, suggesting that, the surfactant molecules preferentially adsorb at the interface prior to micelle formation in water. The small values of ΔS_mic_ and ΔS_ads,_ and the slight decreases of ΔH_mic_ and ΔH_ads_ with rising temperature, may be attributed to the water molecules encasing the alkyl chain. These molecules transferred from the aqueous medium into the inner cavity of the micelle, rendering the ΔG_mic_ and ΔG_ads_ more –ve by about 2.5kj/10°C.

The results demonstrated that, the prepared surfactants are recommended to be used for increasing the oil production in petroleum industry.

## Supplementary Information

Supplementary Information 1.

Supplementary Information 2.

Supplementary Information 3.

Supplementary Information 4.

Supplementary Information 5.

Supplementary Information 6.

Supplementary Information 7.

Supplementary Information 8.

Supplementary Information 9.

Supplementary Information 10.

Supplementary Information 11.

## Data Availability

Most data generated or analyzed during this study are included in this published article and its supplementary information files. Any other data will be available from the corresponding author upon reasonable request.
