# Supplementary figures and images for: Synthesis of new zwitterionic surfactants and investigation of their surface active and thermodynamic properties
Source: Sci Rep. 2025 May 6;15:15737. doi: 10.1038/s41598-025-97814-6 (PMC12053616; doi:10.1038/s41598-025-97814-6)

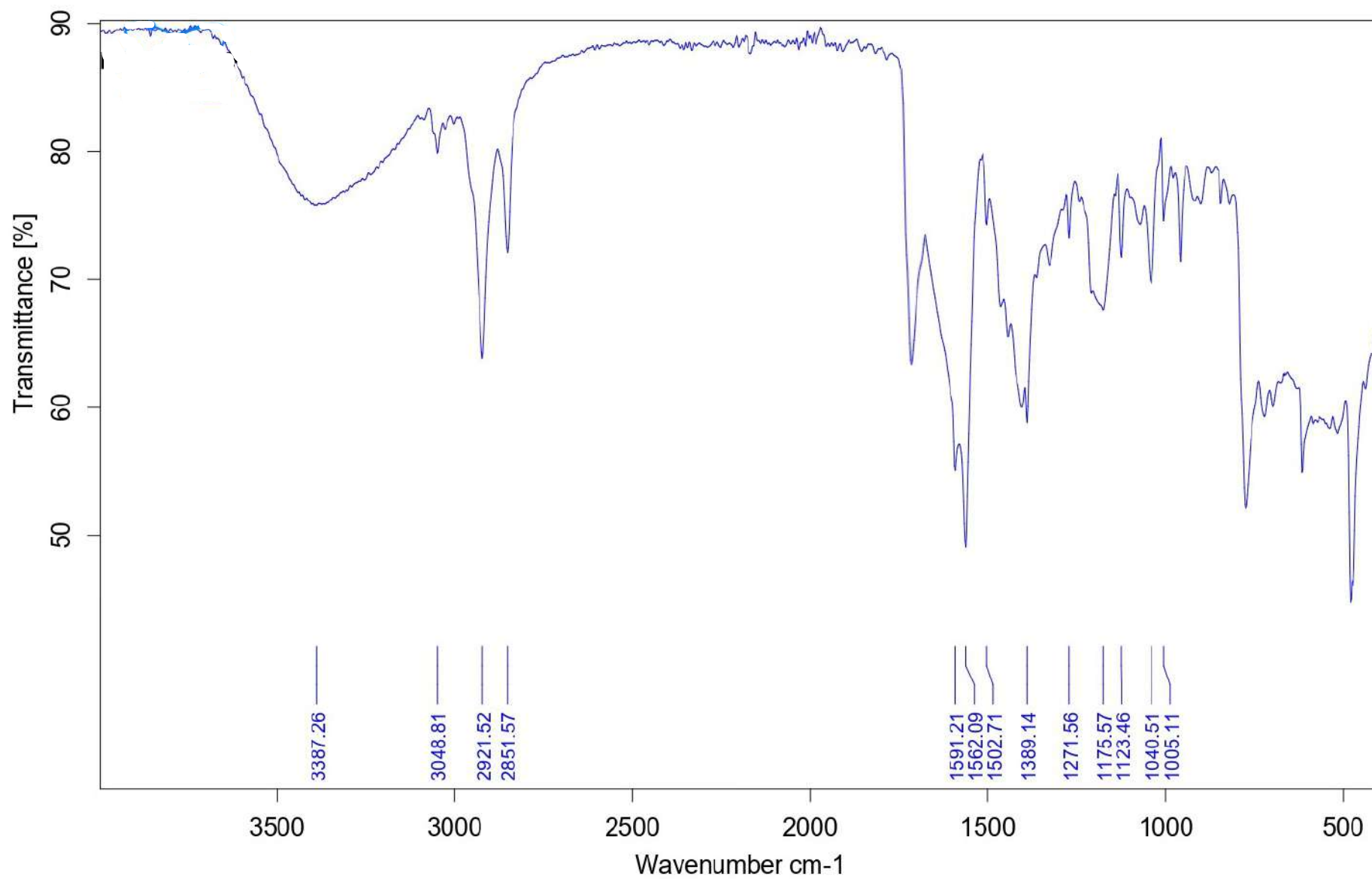

Supplement: Supplementary file 1 — Supplementary Information 1. [file 41598_2025_97814_MOESM1_ESM.pdf]

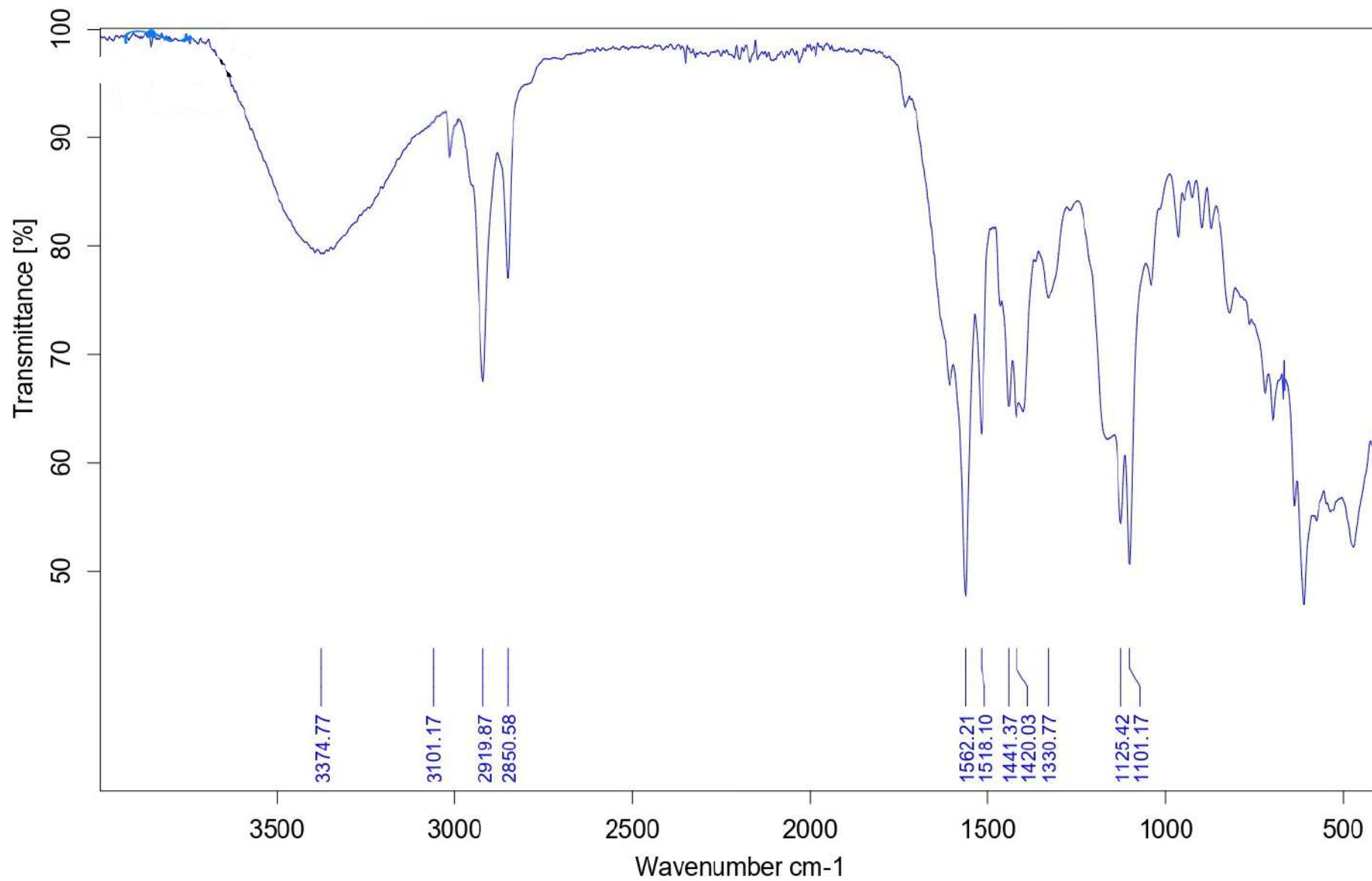

Supplement: Supplementary file 7 — Supplementary Information 7. [file 41598_2025_97814_MOESM7_ESM.pdf]
