# Supplementary material for: Synthesis of new zwitterionic surfactants and investigation of their surface active and thermodynamic properties
Source: Sci Rep. 2025 May 6;15:15737. doi: 10.1038/s41598-025-97814-6 (PMC12053616; doi:10.1038/s41598-025-97814-6)
Supplement: Supplementary file 2 — Supplementary Information 2. [file 41598_2025_97814_MOESM2_ESM.pdf]

وزارة البحث العلمى
معهد بحوث البترول المصرى
Egyptian Petroleum Research Institute (EPRI)
وحدة الاستخلاص المحسن للنفط
Enhanced Oil Recovery (EOR)
ISO 17025, 14001, 18001, 9001

Results of Surface Tension and Interfacial Tension of BE, BP, BPh, NE, NP & NPh surfactants of the

PhD student / Ahmed Samy Mansour

President of EOR Project

Prof. ( )

١ ش أحمد الزمر - مدينة نصر - القاهرة تليفون : ٢٢٧٤٧٩١٧ - ٢٢٧٤٧٨٤٧ (+٢٠٢) فاكس : ٢٢٧٤٧٤٣٣ (+٢٠٢) الرقم البريدى : ١١٧٢٧
1, Ahmed El-Zomor St., Nasr City, Cairo, Egypt. Tel: (+202) 22747917 / 22747847 Fax: (+202) 22747433 P.O.: 11727
www.epri.sci.eg E-mail: research@epri.sci.eg

SURFACE TENTION MEASURMENTS OF SURFACTANT BP/ Ahmed Samy

Sur./conc.	Conc. (gm/100ml)	Conc. x 10	(Conc. x 10)/M.wt.	ln ((Conc. x 10)/M.wt.)	γmN/m (±0.5)			
					60°C	50°C	40°C	30°C
BP/0.625ppm	0.0000625	0.000625	8.62069E-07	-13.96393056	61	63	65	67
BP/1.25ppm	0.000125	0.00125	1.72414E-06	-13.27078338	58	61	64	66
BP/2.5ppm	0.00025	0.0025	3.44828E-06	-12.5776362	56	58	61	63
BP/5ppm	0.0005	0.005	6.89655E-06	-11.88448902	53	55	57	61
BP/10ppm	0.001	0.01	1.37931E-05	-11.19134184	50	52	54	58
BP/25ppm	0.0025	0.025	3.44828E-05	-10.27505111	45	48	51	54
BP/50ppm	0.005	0.05	6.89655E-05	-9.581903928	43	46	48	52
BP/100ppm	0.01	0.1	0.000137931	-8.888756748	40	43	46	50
BP/200ppm	0.02	0.2	0.000275862	-8.195609567	38	40	44	47
BP/300ppm	0.03	0.3	0.000413793	-7.790144459	36	39	42	45
BP/625ppm	0.0625	0.625	0.000862069	-7.056175284	34	37	40	43
BP/1250ppm	0.125	1.25	0.001724138	-6.363028104	34	35	38	41
BP/2500ppm	0.25	2.5	0.003448276	-5.669880923	34	35	36	38
BP/5000ppm	0.5	5	0.006896552	-4.976733742	34	35	36	37
BP/10000ppm	1	10	0.013793103	-4.283586562	34	35	36	37
BP/20000ppm	2	20	0.027586207	-3.590439381	34	35	36	37
BP/40000ppm	4	40	0.055172414	-2.897292201	34	35	36	37

١ ش احمد الزمر - مدينة نصر - القاهرة تليفون : ٢٢٧٤٧٩١٧ - ٢٢٧٤٧٨٤٧ (+٢٠٢) فاكس : ٢٢٧٤٧٤٣٣ (+٢٠٢) الرقم البريدى : ١١٧٢٧
1, Ahmed El-Zomor St., Nasr City, Cairo, Egypt. Tel: (+202) 22747917 / 22747847 Fax: (+202) 22747433 P.O.: 11727
www.epri.sci.eg E-mail: research@epri.sci.eg

SURFACE TENTION MEASURMENTS OF SURFACTANT BE/Ahmed Samy

Sur./conc.	Conc. (gm/100ml)	Conc. x 10	(Conc. x 10)/M.wt.	ln((Conc. x 10)/M.wt.)	γmN/m (±0.5)			
					60°C	50°C	40°C	30°C
BE/0.625ppm	0.0000625	0.000625	8.967E-07	-13.92454432	58	60	62	65
BE/1.25ppm	0.000125	0.00125	1.7934E-06	-13.23139714	54	56	58	61
BE/2.5ppm	0.00025	0.0025	3.5868E-06	-12.53824996	50	53	55	58
BE/5ppm	0.0005	0.005	7.1736E-06	-11.84510278	48	50	53	55
BE/10ppm	0.001	0.01	1.43472E-05	-11.1519556	46	48	50	53
BE/25ppm	0.0025	0.025	3.5868E-05	-10.23566486	43	45	47	50
BE/50ppm	0.005	0.05	7.1736E-05	-9.542517684	41	43	45	48
BE/100ppm	0.01	0.1	0.000143472	-8.849370504	39	41	43	46
BE/200ppm	0.02	0.2	0.000286944	-8.156223323	37	39	41	44
BE/300ppm	0.03	0.3	0.000430416	-7.750758215	35	37	40	42
BE/625ppm	0.0625	0.625	0.0008967	-7.01678904	32	35	37	40
BE/1250ppm	0.125	1.25	0.0017934	-6.323641859	32	33	35	37
BE/2500ppm	0.25	2.5	0.003586801	-5.630494679	32	33	34	36
BE/5000ppm	0.5	5	0.007173601	-4.937347498	32	33	34	35
BE/10000ppm	1	10	0.014347202	-4.244200318	32	33	34	35
BE/20000ppm	2	20	0.028694405	-3.551053137	32	33	34	35
BE/40000ppm	4	40	0.057388809	-2.857905957	32	33	34	35

١ ش أحمد الزمر - مدينة نصر - القاهرة تليفون : ٢٢٧٤٧٩١٧ - ٢٢٧٤٧٨٤٧ (+٢٠٢) فاكس : ٢٢٧٤٧٤٣٣ (+٢٠٢) الرقم البريدى : ١١٧٢٧
1, Ahmed El-Zomor St., Nasr City, Cairo, Egypt. Tel: (+202) 22747917 / 22747847 Fax: (+202) 22747433 P.O.: 11727
www.epri.sci.eg E-mail: research@epri.sci.eg

وزارة البحث العلمى
معهد بحوث البتـرول المصرى
Egyptian Petroleum Research Institute (EPRI)
وحدة الاستخلاص المحسن للنفط
Enhanced Oil Recovery (EOR)

EGAC Accredited

ISO 17025, 14001, 18001, 9001

SURFACE TENTION MEASURMENTS OF SURFACTANT BPh/ Ahmed Samy

Sur./conc.	Conc. (gm/100ml)	Conc. x 10	(Conc. x 10)/M.wt.	ln ((Conc. x 10)/M.wt.)	γmN/m (±0.5)			
					60°C	50°C	40°C	30°C
BPh/0.625ppm	0.0000625	0.000625	7.88146E-07	-14.05358213	62	64	66	68
BPh/1.25ppm	0.00013	0.00125	1.57629E-06	-13.36043495	59	61	63	65
BPh/2.5ppm	0.00025	0.0025	3.15259E-06	-12.66728777	56	58	60	62
BPh/5ppm	0.0005	0.005	6.30517E-06	-11.97414059	54	56	58	60
Bph/10ppm	0.001	0.01	1.26103E-05	-11.28099341	51	53	55	58
Bph/25ppm	0.0025	0.025	3.15259E-05	-10.36470268	48	50	52	55
Bph/50ppm	0.005	0.05	6.30517E-05	-9.671555495	45	47	50	52
Bph/100ppm	0.01	0.1	0.000126103	-8.978408315	43	45	47	49
Bph/200ppm	0.02	0.2	0.000252207	-8.285261134	40	42	45	47
Bph/300ppm	0.03	0.3	0.00037831	-7.879796026	39	41	43	45
Bph/625ppm	0.0625	0.625	0.000788146	-7.145826851	36	38	40	43
Bph/1250ppm	0.125	1.25	0.001576293	-6.45267967	33	36	38	40
Bph/2500ppm	0.25	2.5	0.003152585	-5.75953249	33	35	37	39
Bph/5000ppm	0.5	5	0.00630517	-5.066385309	33	35	36	38
Bph/10000ppm	1	10	0.01261034	-4.373238129	33	35	36	37
Bph/20000ppm	2	20	0.025220681	-3.680090948	33	35	36	37
Bph/40000ppm	4	40	0.050441362	-2.986943768	33	35	36	37

1, Ahmed El-Zomor St., Nasr City, Cairo, Egypt. Tel: (+202) 22747917 / 22747847 Fax: (+202) 22747433 P.O.: 11727
www.epri.sci.eg E-mail: research@epri.sci.eg

SURFACE TENTION MEASURMENTS OF SURFACTANT NE/ Ahmed Samy

Sur./conc.	Conc. (gm/100ml)	Conc. x 10	(Conc. x 10)/M.wt.	ln((Conc. x 10)/M.wt.)	γmN/m (±0.5)			
					60°C	50°C	40°C	30°C
NE/0.625 ppm	0.0000625	0.000625	8.3668E-07	-13.99382409	62	64	66	68
NE/1.25 ppm	0.000125	0.00125	1.67336E-06	-13.30067691	60	62	64	66
NE/2.5 ppm	0.00025	0.0025	3.34672E-06	-12.60752973	57	59	60	62
NE/5 ppm	0.0005	0.005	6.69344E-06	-11.91438255	53	55	57	59
NE/10 ppm	0.001	0.01	1.33869E-05	-11.22123537	49	52	54	56
NE/25 ppm	0.0025	0.025	3.34672E-05	-10.30494464	46	48	50	52
NE/50 ppm	0.005	0.05	6.69344E-05	-9.611797459	43	45	47	49
NE/100 ppm	0.01	0.1	0.000133869	-8.918650278	41	42	45	47
NE/200 ppm	0.02	0.2	0.000267738	-8.225503098	38	40	43	45
NE/300 ppm	0.03	0.3	0.000401606	-7.820037989	37	39	42	44
NE/625 ppm	0.0625	0.625	0.00083668	-7.086068814	35	37	40	42
NE/1250 ppm	0.125	1.25	0.00167336	-6.392921634	35	36	38	41
NE/2500 ppm	0.25	2.5	0.00334672	-5.699774453	35	36	37	40
NE/5000 ppm	0.5	5	0.00669344	-5.006627273	35	36	37	39
NE/10000 ppm	1	10	0.013386881	-4.313480092	35	36	37	39
NE/20000 PPM	2	20	0.026773762	-3.620332912	35	36	37	39
NE/40000 PPM	4	40	0.053547523	-2.927185731	35	36	37	39

١ ش احمد الزمر - مدينة نصر - القاهرة تليفون : ٢٢٧٤٧٩١٧ - ٢٢٧٤٧٨٤٧ (٢٠٢+) فاكس : ٢٢٧٤٧٤٣٣ (٢٠٢+) الرقم البريدى : ١١٧٢٧
1, Ahmed El-Zomor St., Nasr City, Cairo, Egypt. Tel: (+202) 22747917 / 22747847 Fax: (+202) 22747433 P.O.: 11727
www.epri.sci.eg E-mail: research@epri.sci.eg

SURFACE TENTION MEASURMENTS OF SURFACTANT NP/ Ahmed Samy

Sur./conc.	Conc. (gm/100ml)	Conc. x 10	(Conc. x 10)/M.wt.	ln ((Conc. x 10)/M.wt.)	γmN/m (±0.5)			
					60°C	50°C	40°C	30°C
NP/0.625 ppm	0.0000625	0.000625	8.06452E-07	-14.03062194	62	64	66	68
NP/1.25 ppm	0.000125	0.00125	1.6129E-06	-13.33747476	60	62	64	66
NP/2.5 ppm	0.00025	0.0025	3.22581E-06	-12.64432758	57	58	60	62
NP/5 ppm	0.0005	0.005	6.45161E-06	-11.9511804	54	56	57	60
NP/10 ppm	0.001	0.01	1.29032E-05	-11.25803322	51	53	55	58
NP/25 ppm	0.0025	0.025	3.22581E-05	-10.34174248	48	50	52	56
NP/50 ppm	0.005	0.05	6.45161E-05	-9.648595303	45	47	50	53
NP/100 ppm	0.01	0.1	0.000129032	-8.955448122	42	44	48	50
NP/200 ppm	0.02	0.2	0.000258065	-8.262300942	40	42	45	48
NP/300 ppm	0.03	0.3	0.000387097	-7.856835834	39	41	43	46
NP/625 ppm	0.0625	0.625	0.000806452	-7.122866659	37	39	41	44
NP/1250 ppm	0.125	1.25	0.001612903	-6.429719478	35	37	39	42
NP/2500 ppm	0.25	2.5	0.003225806	-5.736572297	35	36	38	40
NP/5000 ppm	0.5	5	0.006451613	-5.043425117	35	36	37	39
NP/10000 ppm	1	10	0.012903226	-4.350277936	35	36	37	38
NP/20000 ppm	2	20	0.025806452	-3.657130756	35	36	37	38
NP/40000 ppm	4	40	0.051612903	-2.963983575	35	36	37	38

١ ش احمد الزمر - مدينة نصر - القاهرة تليفون : ٢٢٧٤٧٩١٧ - ٢٢٧٤٧٨٤٧ (٢٠٢+) فاكس : ٢٢٧٤٧٤٣٣ (٢٠٢+) الرقم البريدى : ١١٧٢٧
1, Ahmed El-Zomor St., Nasr City, Cairo, Egypt. Tel: (+202) 22747917 / 22747847 Fax: (+202) 22747433 P.O.: 11727
www.epri.sci.eg E-mail: research@epri.sci.eg

وزارة البحث العلمى
معهد بحوث البترول المصرى
Egyptian Petroleum Research Institute (EPRI)
وحدة الاستخلاص المحسن للنفط
Enhanced Oil Recovery (EOR)
ISO 17025, 14001, 18001, 9001

SURFACE TENTION MEASURMENTS OF SURFACTANT NPh/ Ahmed Samy

Sur./conc.	Conc. (gm/100ml)	Conc. x 10	(Conc. x 10)/M.wt.	ln ((Conc. x 10)/M.wt.)	γmN/m (±0.5)			
					60°C	50°C	40°C	30°C
NPh/0.625 ppm	0.0000625	0.000625	7.414E-07	-14.11472587	60	62	64	66
NPh/1.25 ppm	0.000125	0.00125	1.4828E-06	-13.42157869	58	60	62	64
NPh/2.5 ppm	0.00025	0.0025	2.9656E-06	-12.72843151	55	57	59	62
NPh/5 ppm	0.0005	0.005	5.9312E-06	-12.03528432	52	54	56	58
NPh/10 ppm	0.001	0.01	1.18624E-05	-11.34213714	48	50	52	54
NPh/25 ppm	0.0025	0.025	2.9656E-05	-10.42584641	45	47	49	51
NPh/50 ppm	0.005	0.05	5.9312E-05	-9.732699232	41	44	46	48
NPh/100 ppm	0.01	0.1	0.000118624	-9.039552051	38	41	43	45
NPh/200 ppm	0.02	0.2	0.000237248	-8.34640487	35	37	40	42
NPh/300 ppm	0.03	0.3	0.000355872	-7.940939762	32	34	37	39
NPh/625 ppm	0.0625	0.625	0.0007414	-7.206970587	30	32	34	36
NPh/1250 ppm	0.125	1.25	0.0014828	-6.513823407	30	31	33	35
NPh/2500 ppm	0.25	2.5	0.002965599	-5.820676226	30	31	32	34
NPh/5000 ppm	0.5	5	0.005931198	-5.127529046	30	31	32	33
NPh/10000 ppm	1	10	0.011862396	-4.434381865	30	31	32	33
NPh/20000 ppm	2	20	0.023724792	-3.741234684	30	31	32	33
NPh/40000 ppm	4	40	0.047449585	-3.048087504	30	31	32	33

١ ش أحمد الزمر - مدينة نصر - القاهرة تليفون : ٢٢٧٤٧٩١٧ - ٢٢٧٤٧٨٤٧ (٢٠٢+) فاكس : ٢٢٧٤٧٤٣٣ (٢٠٢+) الرقم البريدى : ١١٧٢٧
1, Ahmed El-Zomor St., Nasr City, Cairo, Egypt. Tel: (+202) 22747917 / 22747847 Fax: (+202) 22747433 P.O.: 11727
www.epri.sci.eg E-mail: research@epri.sci.eg

INTERFACIAL TENSION MEASURMENTS OF SURFACTANTS BE, BP, BPh, NE, NP&NPh / Ahmed Samy

Surfactant	IFT, mNm^{-1} (± 0.05)	
	50°C	70°C
BE	$4*10^{-1}$	$8*10^{-2}$
BP	$2*10^{-1}$	$6*10^{-2}$
BPh	$3*10^{-1}$	$7*10^{-2}$
NE	$2*10^{-2}$	$< 10^{-2}$
NP	$4*10^{-2}$	$< 10^{-2}$
NPh	$3*10^{-2}$	$< 10^{-2}$

EPRI EOR

١ ش احمد الزمر - مدينة نصر - القاهرة تليفون : ٢٢٧٤٧٩١٧ - ٢٢٧٤٧٨٤٧ (٢٠٢+) فاكس : ٢٢٧٤٧٤٣٣ (٢٠٢+) الرقم البريدى : ١١٧٢٧
1, Ahmed El-Zomor St., Nasr City, Cairo, Egypt. Tel: (+202) 22747917 / 22747847 Fax: (+202) 22747433 P.O.: 11727
www.epri.sci.eg E-mail: research@epri.sci.eg
