# Supplementary material for: Synthesis of new zwitterionic surfactants and investigation of their surface active and thermodynamic properties
Source: Sci Rep. 2025 May 6;15:15737. doi: 10.1038/s41598-025-97814-6 (PMC12053616; doi:10.1038/s41598-025-97814-6)
Supplement: Supplementary file 3 — Supplementary Information 3. [file 41598_2025_97814_MOESM3_ESM.pdf]

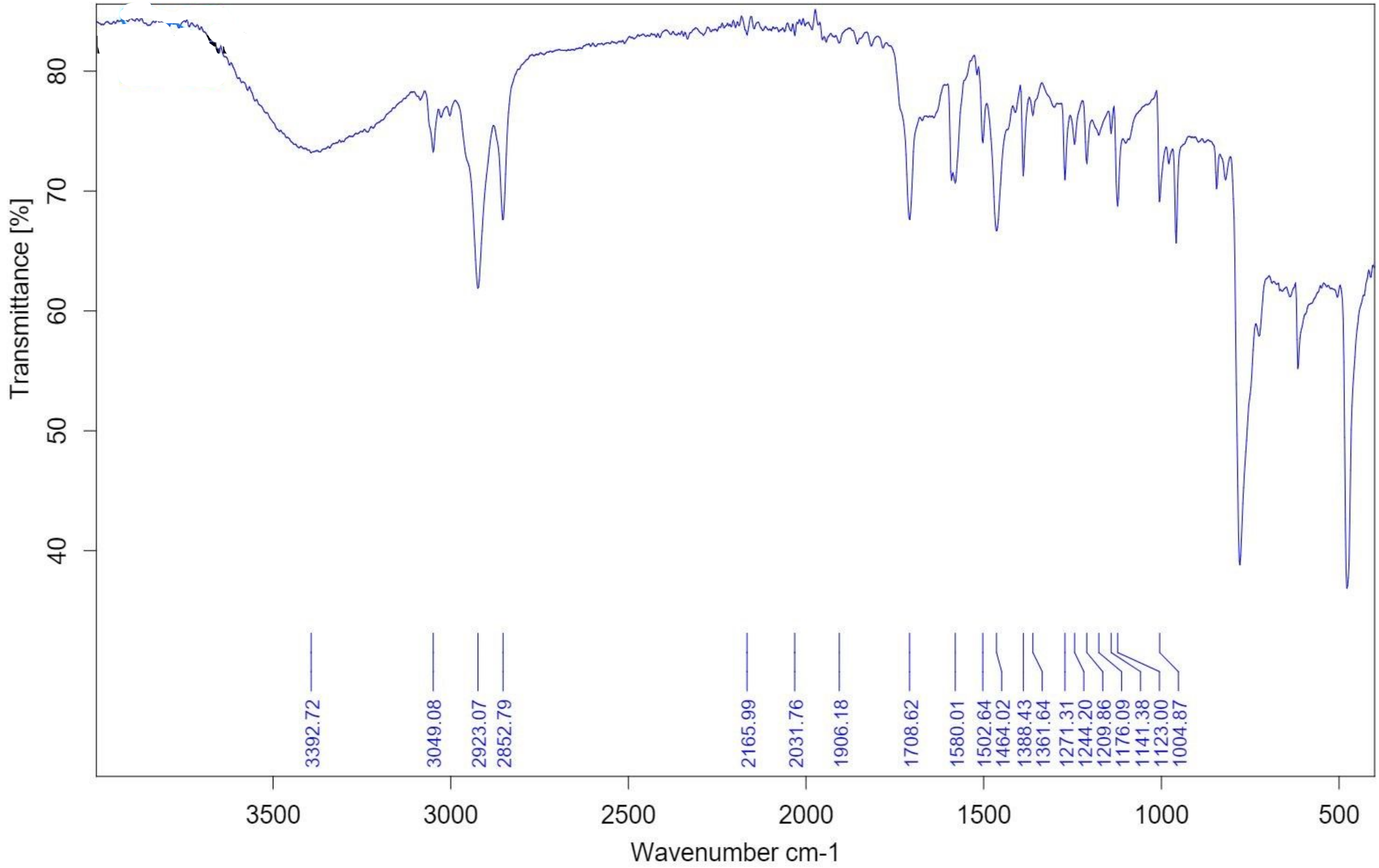

C:\Users\Public\Documents\Bruker\OPUS_8.1.29\DATA\MEAS\Jasmine multiflorum.15	Jasmine multiflorum	Instrument type and / or acce	11/23/2023
