# Supplementary material for: Synthesis of new zwitterionic surfactants and investigation of their surface active and thermodynamic properties
Source: Sci Rep. 2025 May 6;15:15737. doi: 10.1038/s41598-025-97814-6 (PMC12053616; doi:10.1038/s41598-025-97814-6)
Supplement: Supplementary file 4 — Supplementary Information 4. [file 41598_2025_97814_MOESM4_ESM.pdf]

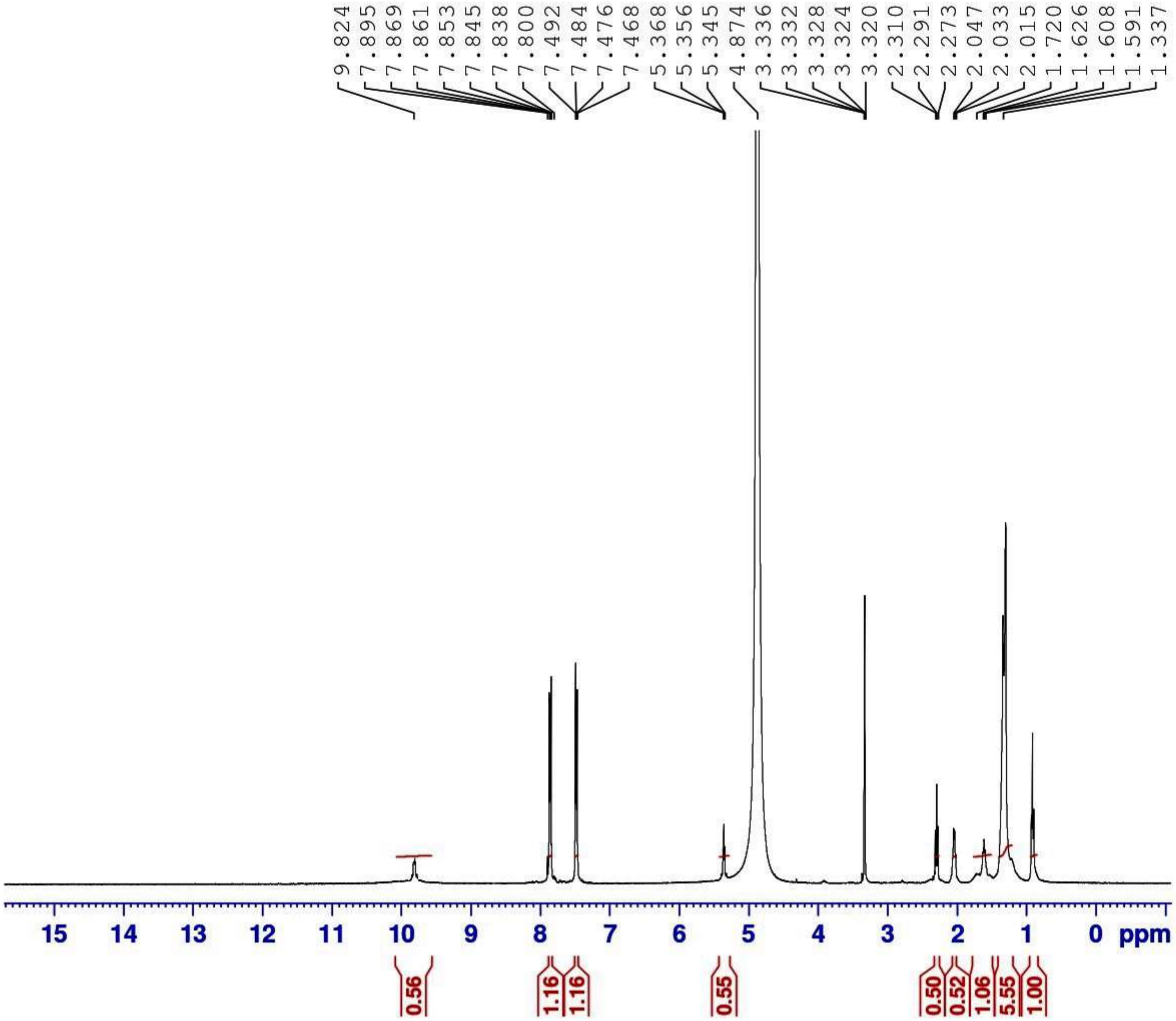

Current Data Parameters

NAME	ahmed-samy-AL-N
EXPNO	1
PROCNO	1

F2 - Acquisition Parameters

Date_	20231127
Time	15.00
INSTRUM	spect
PROBHD	5 mm PABBO BB/
PULPROG	zg30
TD	65536
SOLVENT	MeOD
NS	55
DS	2
SWH	8012.820 Hz
FIDRES	0.122266 Hz
AQ	4.0894465 sec
RG	141.56
DW	62.400 usec
DE	6.50 usec
TE	300.0 K
D1	1.00000000 sec
TD0	1

======== CHANNEL f1 ========

SFO1	400.1524711 MHz
NUC1	1H
P1	12.00 usec
PLW1	18.00000000 W

F2 - Processing parameters

SI	65536
SF	400.1500000 MHz
WDW	EM
SSB	0
LB	0.30 Hz
GB	0
PC	1.00
