# Supplementary material for: Synthesis of new zwitterionic surfactants and investigation of their surface active and thermodynamic properties
Source: Sci Rep. 2025 May 6;15:15737. doi: 10.1038/s41598-025-97814-6 (PMC12053616; doi:10.1038/s41598-025-97814-6)
Supplement: Supplementary file 5 — Supplementary Information 5. [file 41598_2025_97814_MOESM5_ESM.pdf]

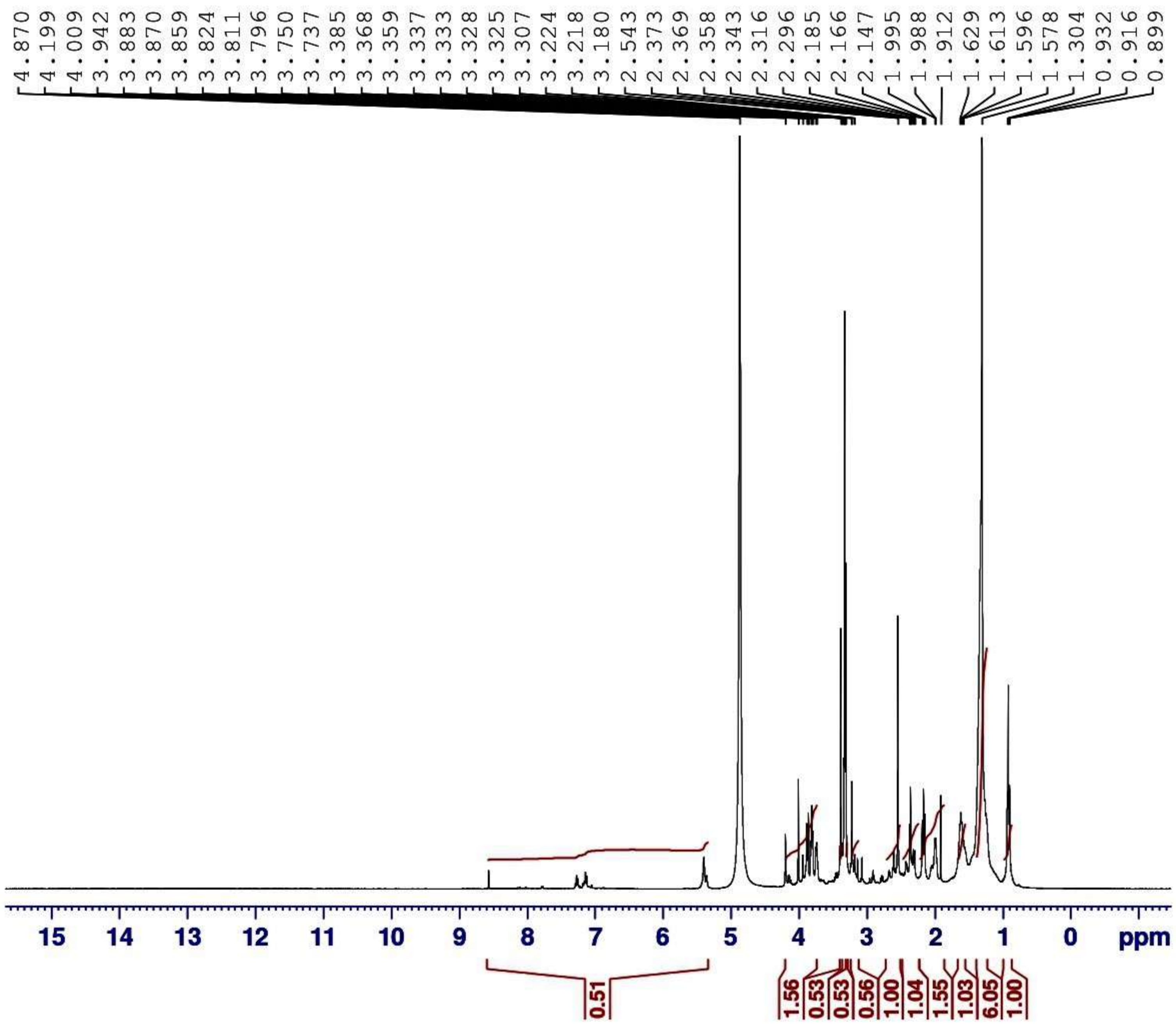

Current Data Parameters
NAME ahmed-samy-BE
EXPNO 1
PROCNO 1

F2 - Acquisition Parameters
Date_ 20231128
Time 17.24
INSTRUM spect
PROBHD 5 mm PABBO BB/
PULPROG zg30
TD 65536
SOLVENT MeOD
NS 128
DS 2
SWH 8012.820 Hz
FIDRES 0.122266 Hz
AQ 4.0894465 sec
RG 205.37
DW 62.400 usec
DE 6.50 usec
TE 300.0 K
D1 1.00000000 sec
TD0 1

======== CHANNEL f1 ========
SFO1 400.1524711 MHz
NUC1 1H
P1 12.00 usec
PLW1 18.00000000 W

F2 - Processing parameters
SI 65536
SF 400.1500000 MHz
WDW EM
SSB 0
LB 0.30 Hz
GB 0
PC 1.00

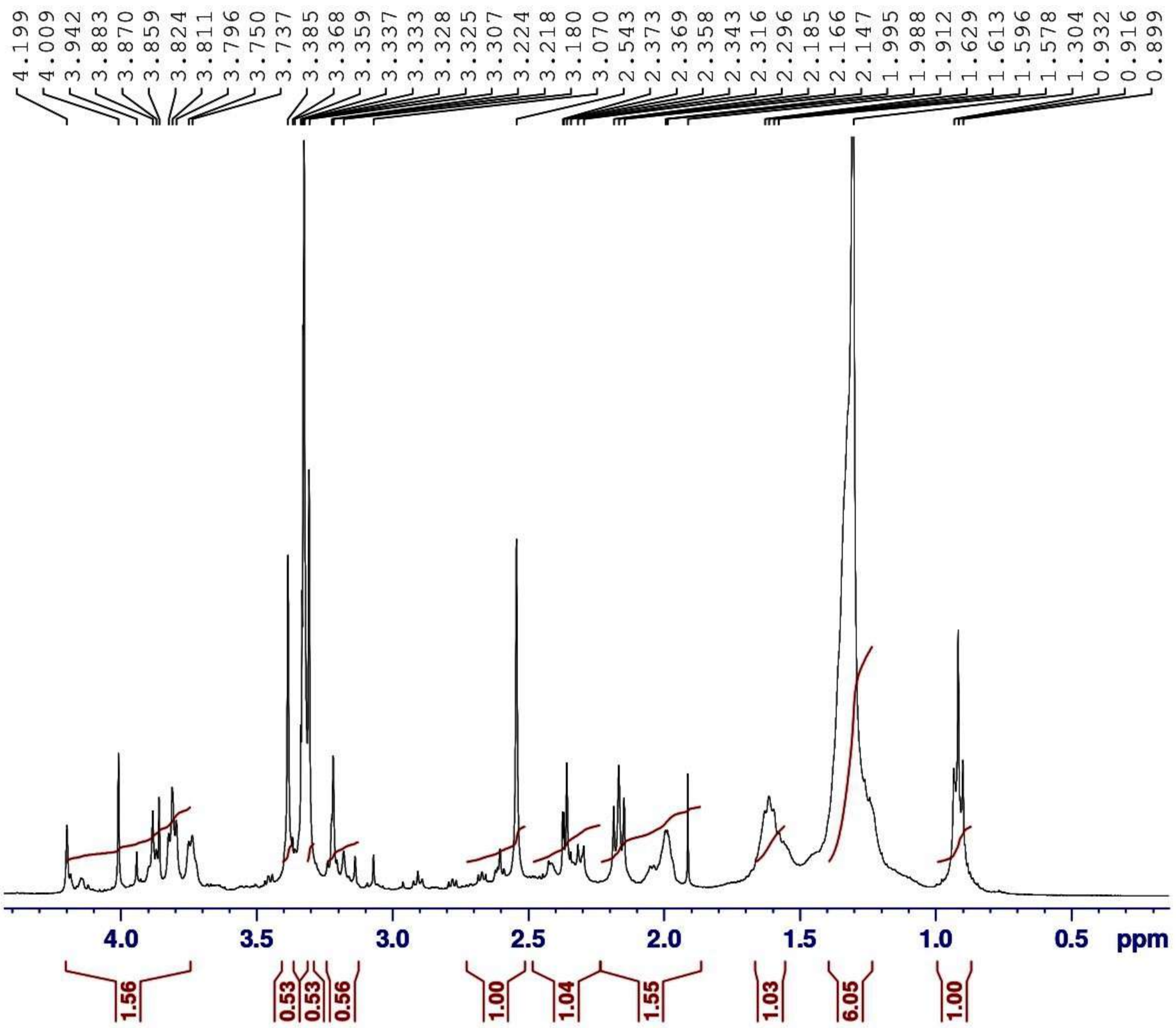

Current Data Parameters
NAME ahmed-samy-BE
EXPNO 1
PROCNO 1

F2 - Acquisition Parameters
Date_ 20231128
Time 17.24
INSTRUM spect
PROBHD 5 mm PABBO BB/
PULPROG zg30
TD 65536
SOLVENT MeOD
NS 128
DS 2
SWH 8012.820 Hz
FIDRES 0.122266 Hz
AQ 4.0894465 sec
RG 205.37
DW 62.400 usec
DE 6.50 usec
TE 300.0 K
D1 1.00000000 sec
TD0 1

======== CHANNEL f1 ========
SFO1 400.1524711 MHz
NUC1 1H
P1 12.00 usec
PLW1 18.00000000 W

F2 - Processing parameters
SI 65536
SF 400.1500000 MHz
WDW EM
SSB 0
LB 0.30 Hz
GB 0
PC 1.00
