# Supplementary material for: Synthesis of new zwitterionic surfactants and investigation of their surface active and thermodynamic properties
Source: Sci Rep. 2025 May 6;15:15737. doi: 10.1038/s41598-025-97814-6 (PMC12053616; doi:10.1038/s41598-025-97814-6)
Supplement: Supplementary file 6 — Supplementary Information 6. [file 41598_2025_97814_MOESM6_ESM.pdf]

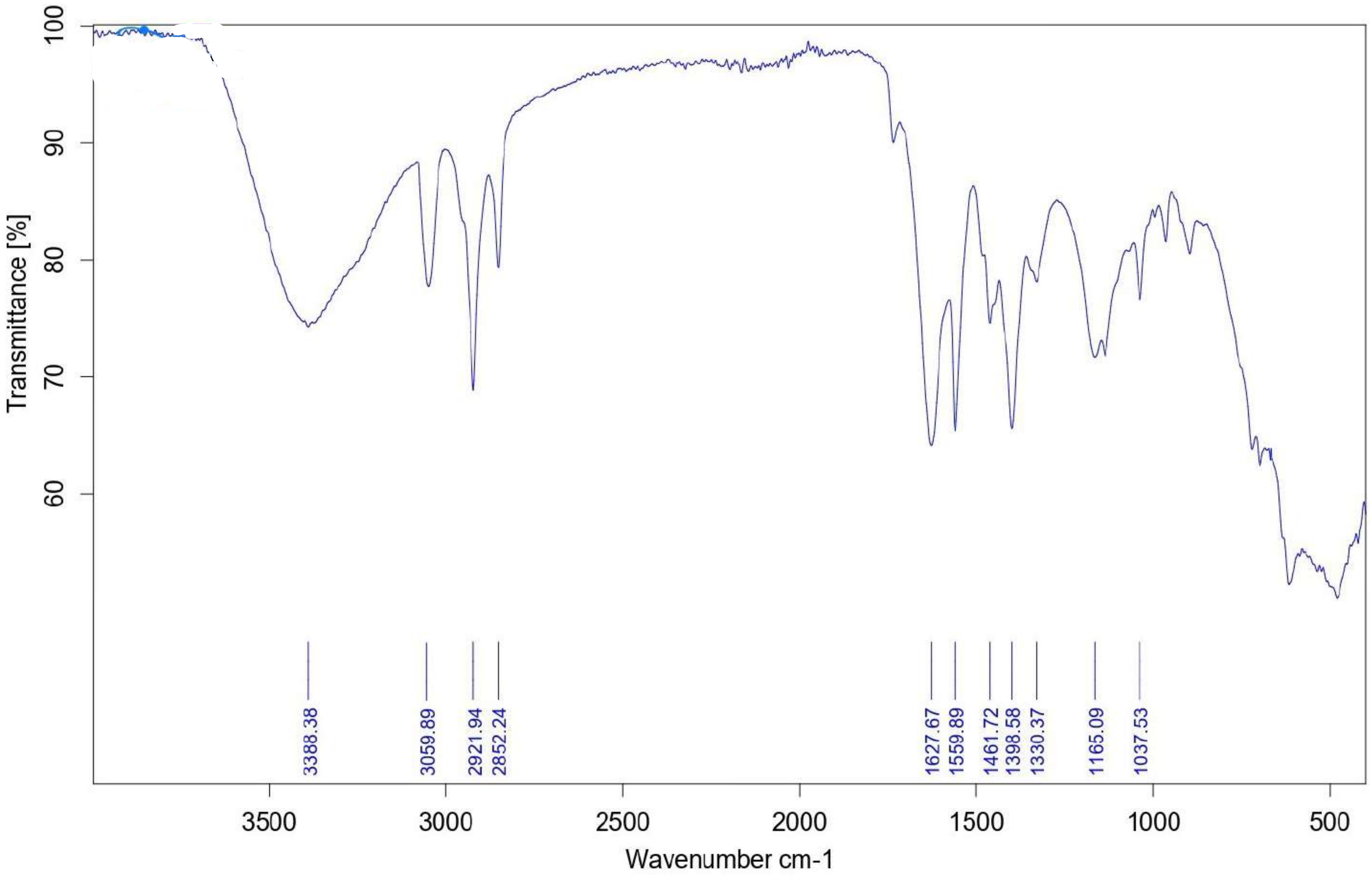


| | |
|---|---|
| C:\Users\Public\Documents\Bruker\OPUS_8.1.29\DATA\MEAS\Jasmine multiflorum.10 Jasmine multiflorum Instrument type and / or acce | 11/23/2023 |
