# Supplementary material for: Synthesis of new zwitterionic surfactants and investigation of their surface active and thermodynamic properties
Source: Sci Rep. 2025 May 6;15:15737. doi: 10.1038/s41598-025-97814-6 (PMC12053616; doi:10.1038/s41598-025-97814-6)
Supplement: Supplementary file 8 — Supplementary Information 8. [file 41598_2025_97814_MOESM8_ESM.pdf]

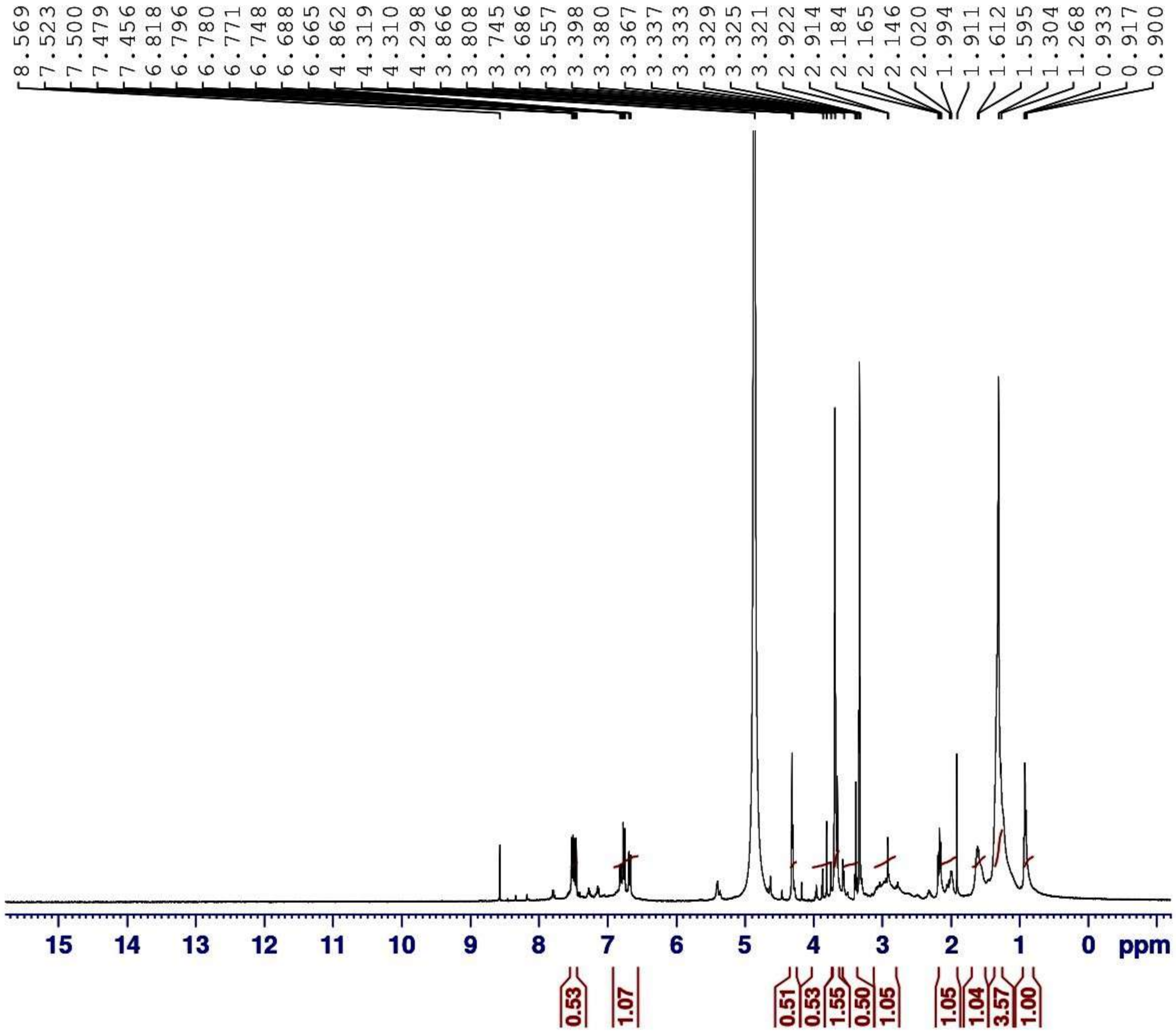

Current Data Parameters
NAME ahmed-samy-BPH
EXPNO 1
PROCNO 1

F2 - Acquisition Parameters
Date_ 20231128
Time 16.52
INSTRUM spect
PROBHD 5 mm PABBO BB/
PULPROG zg30
TD 65536
SOLVENT MeOD
NS 88
DS 2
SWH 8012.820 Hz
FIDRES 0.122266 Hz
AQ 4.0894465 sec
RG 205.37
DW 62.400 usec
DE 6.50 usec
TE 300.0 K
D1 1.00000000 sec
TD0 1

======== CHANNEL f1 ========
SFO1 400.1524711 MHz
NUC1 1H
P1 12.00 usec
PLW1 18.00000000 W

F2 - Processing parameters
SI 65536
SF 400.1500000 MHz
WDW EM
SSB 0
LB 0.30 Hz
GB 0
PC 1.00

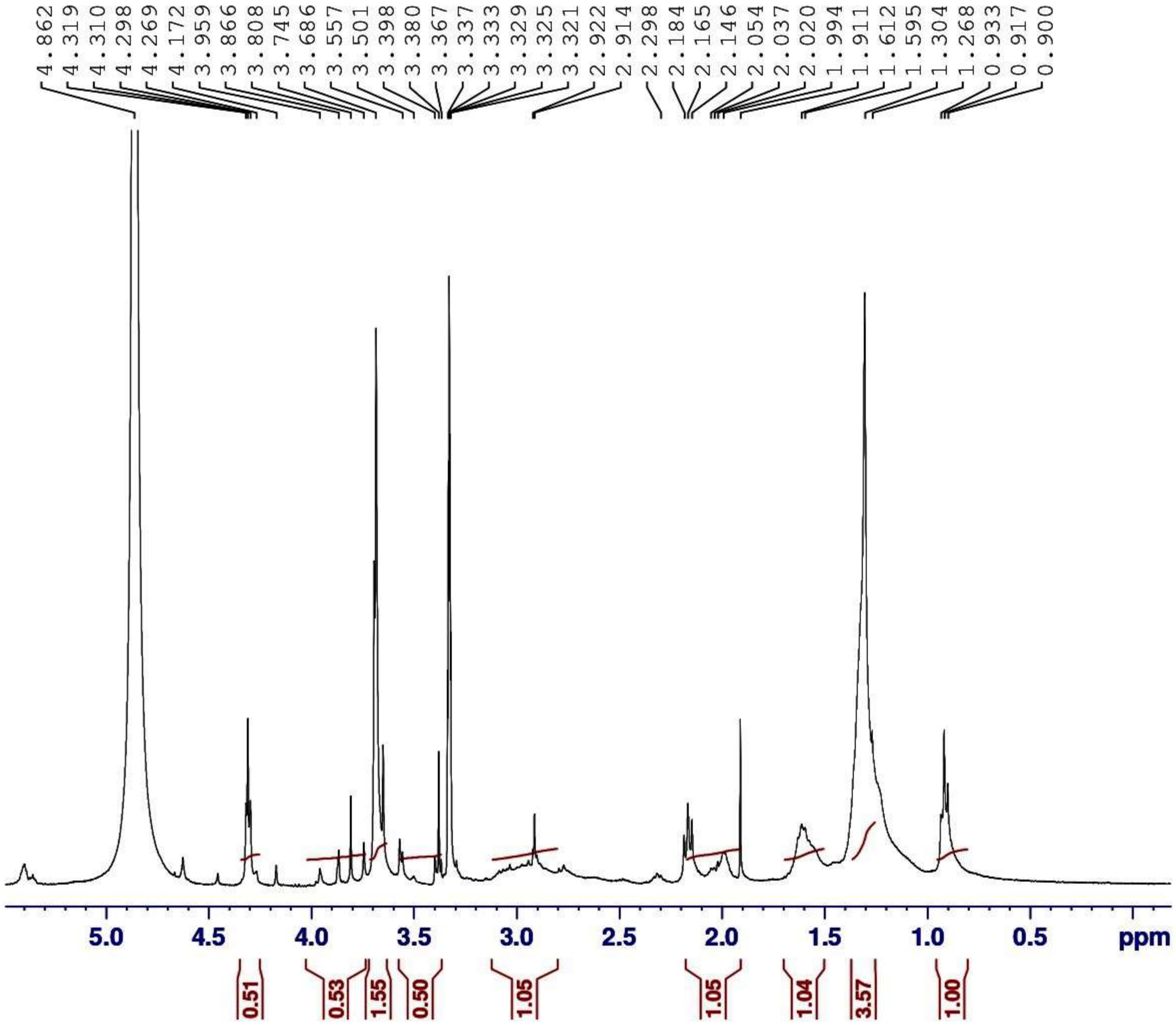

Current Data Parameters
NAME ahmed-samy-BPH
EXPNO 1
PROCNO 1

F2 - Acquisition Parameters

Date_	20231128	
Time	16.52	
INSTRUM	spect	
PROBHD	5 mm PABBO BB/	
PULPROG	zg30	
TD	65536	
SOLVENT	MeOD	
NS	88	
DS	2	
SWH	8012.820	Hz
FIDRES	0.122266	Hz
AQ	4.0894465	sec
RG	205.37	
DW	62.400	usec
DE	6.50	usec
TE	300.0	K
D1	1.00000000	sec
TD0	1	

======== CHANNEL f1 ========

SFO1	400.1524711	MHz
NUC1	1H	
P1	12.00	usec
PLW1	18.00000000	W

F2 - Processing parameters

SI	65536	
SF	400.1500000	MHz
WDW	EM	
SSB	0	
LB	0.30	Hz
GB	0	
PC	1.00	

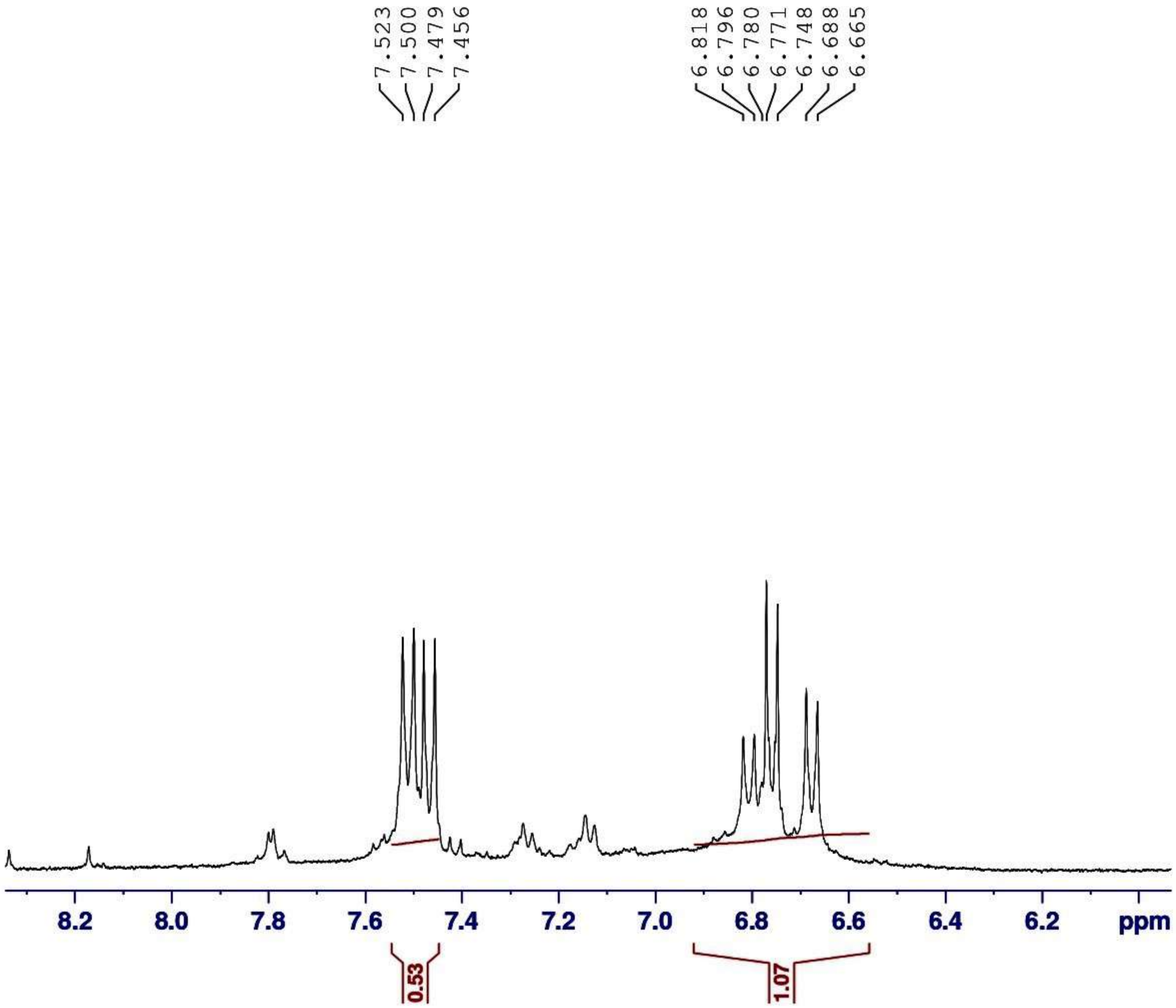

Current Data Parameters
NAME ahmed-samy-BPH
EXPNO 1
PROCNO 1

F2 - Acquisition Parameters
Date_ 20231128
Time 16.52
INSTRUM spect
PROBHD 5 mm PABBO BB/
PULPROG zg30
TD 65536
SOLVENT MeOD
NS 88
DS 2
SWH 8012.820 Hz
FIDRES 0.122266 Hz
AQ 4.0894465 sec
RG 205.37
DW 62.400 usec
DE 6.50 usec
TE 300.0 K
D1 1.00000000 sec
TD0 1

======== CHANNEL f1 ========
SFO1 400.1524711 MHz
NUC1 1H
P1 12.00 usec
PLW1 18.00000000 W

F2 - Processing parameters
SI 65536
SF 400.1500000 MHz
WDW EM
SSB 0
LB 0.30 Hz
GB 0
PC 1.00
