# Supplementary material for: Synthesis of new zwitterionic surfactants and investigation of their surface active and thermodynamic properties
Source: Sci Rep. 2025 May 6;15:15737. doi: 10.1038/s41598-025-97814-6 (PMC12053616; doi:10.1038/s41598-025-97814-6)
Supplement: Supplementary file 9 — Supplementary Information 9. [file 41598_2025_97814_MOESM9_ESM.pdf]

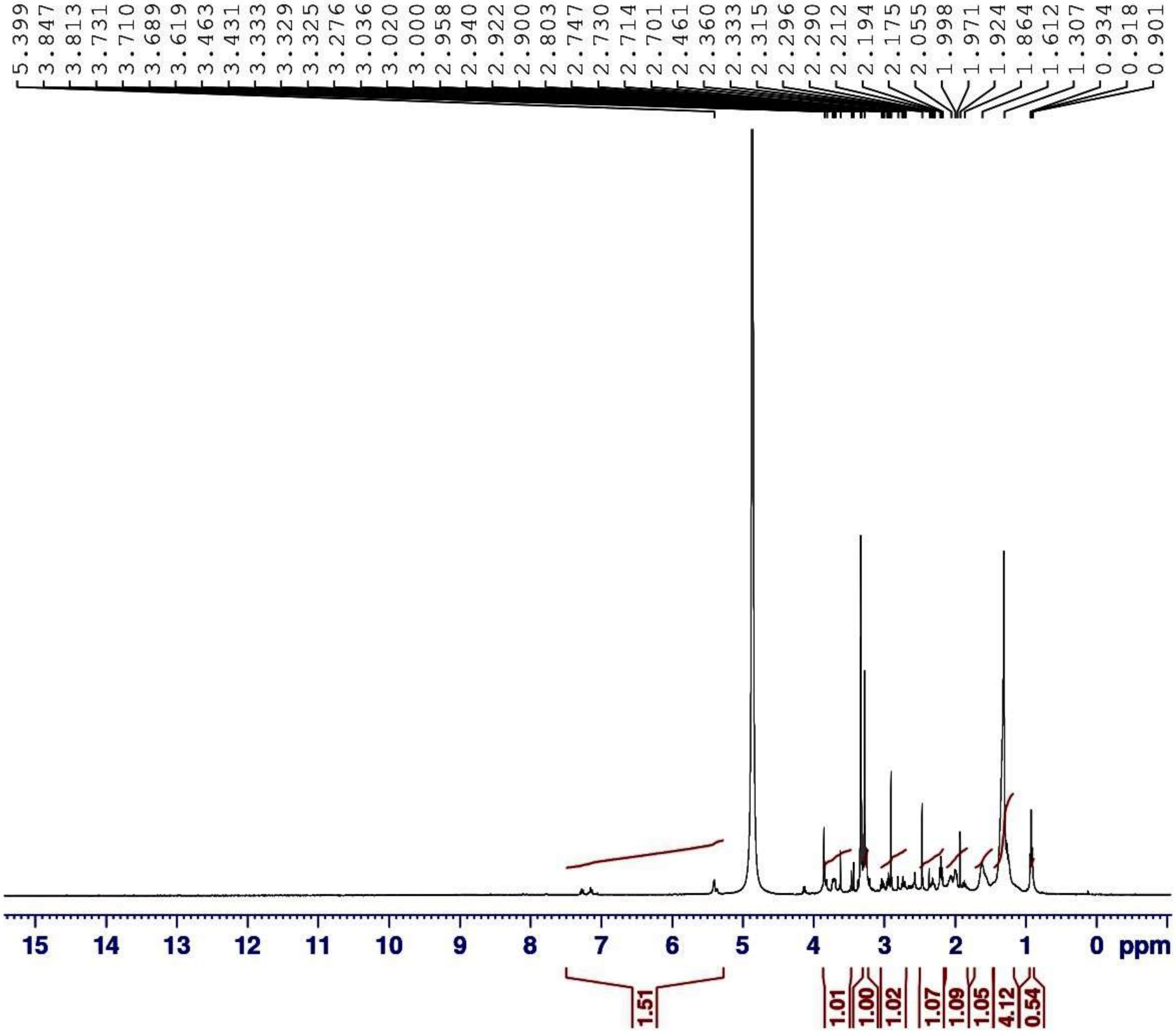

Current Data Parameters

NAME	ahmed-samy-BP
EXPNO	1
PROCNO	1

F2 - Acquisition Parameters

Date_	20231128
Time	17.02
INSTRUM	spect
PROBHD	5 mm PABBO BB/
PULPROG	zg30
TD	65536
SOLVENT	MeOD
NS	83
DS	2
SWH	8012.820 Hz
FIDRES	0.122266 Hz
AQ	4.0894465 sec
RG	205.37
DW	62.400 usec
DE	6.50 usec
TE	300.0 K
D1	1.00000000 sec
TD0	1

======== CHANNEL f1 ========

SFO1	400.1524711 MHz
NUC1	1H
P1	12.00 usec
PLW1	18.00000000 W

F2 - Processing parameters

SI	65536
SF	400.1500000 MHz
WDW	EM
SSB	0
LB	0.30 Hz
GB	0
PC	1.00

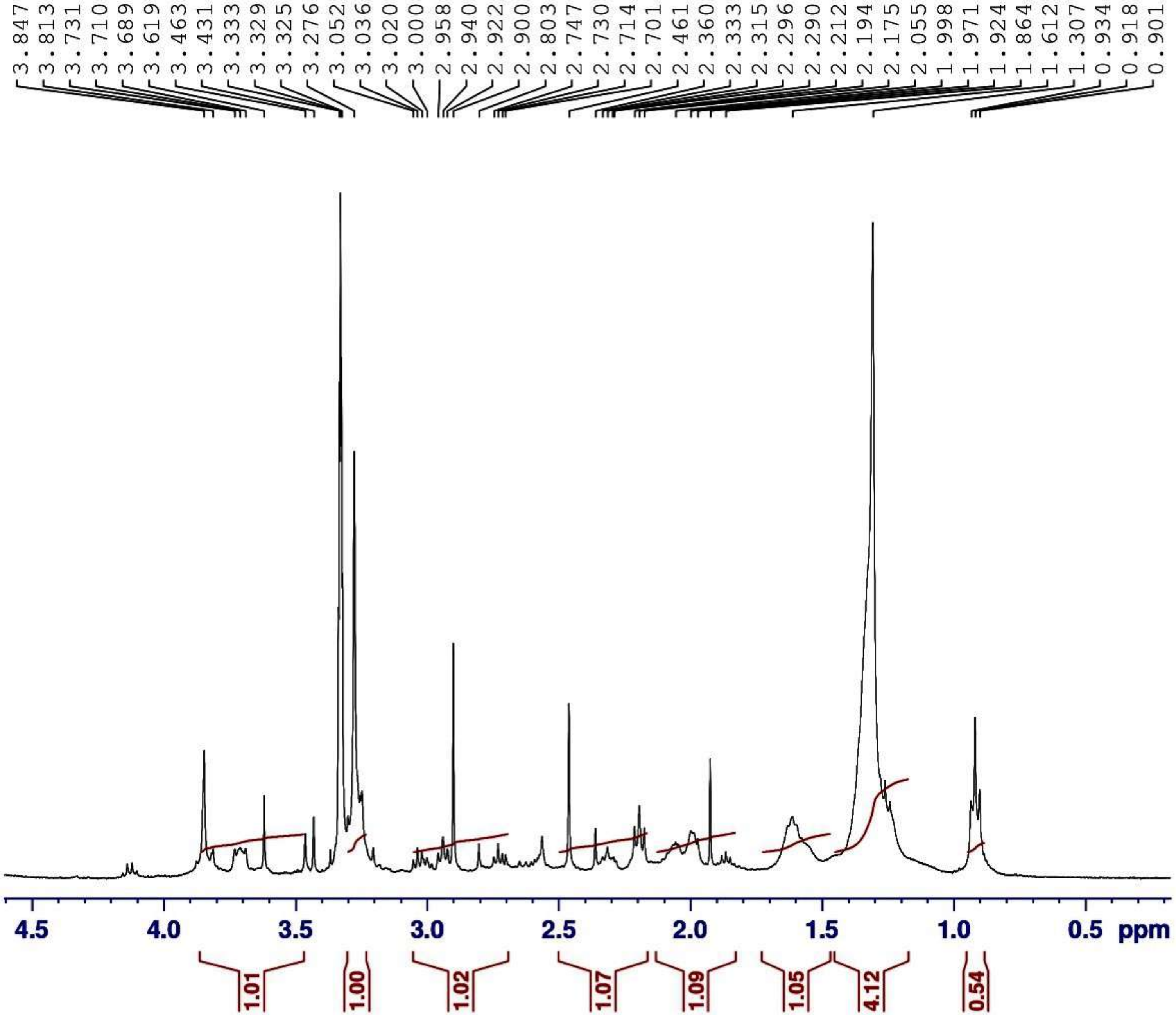


```
Current Data Parameters
NAME          ahmed-samy-BP
EXPNO                     1
PROCNO                    1

F2 - Acquisition Parameters
Date_              20231128
Time                  17.02
INSTRUM               spect
PROBHD      5 mm PABBO BB/
PULPROG                zg30
TD                    65536
SOLVENT                MeOD
NS                       83
DS                        2
SWH              8012.820 Hz
FIDRES           0.122266 Hz
AQ              4.0894465 sec
RG                   205.37
DW                 62.400 usec
DE                   6.50 usec
TE                  300.0 K
D1             1.00000000 sec
TD0                       1

======== CHANNEL f1 ========
SFO1          400.1524711 MHz
NUC1                     1H
P1                  12.00 usec
PLW1          18.00000000 W

F2 - Processing parameters
SI                    65536
SF            400.1500000 MHz
WDW                      EM
SSB          0
LB                     0.30 Hz
GB           0
PC                     1.00
```
