# Supplementary material for: Synthesis of new zwitterionic surfactants and investigation of their surface active and thermodynamic properties
Source: Sci Rep. 2025 May 6;15:15737. doi: 10.1038/s41598-025-97814-6 (PMC12053616; doi:10.1038/s41598-025-97814-6)
Supplement: Supplementary file 11 — Supplementary Information 11. [file 41598_2025_97814_MOESM11_ESM.pdf]

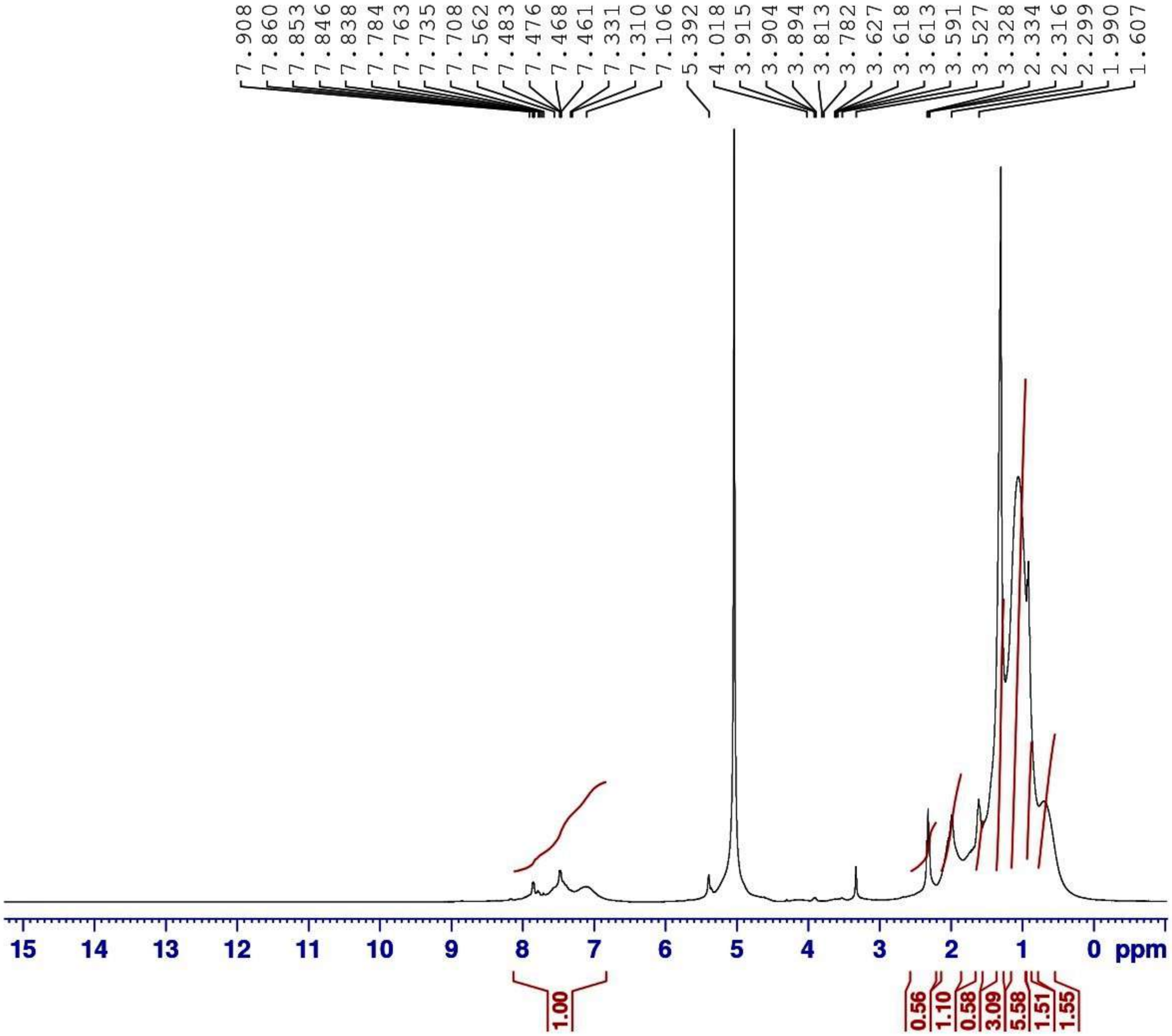


```
Current Data Parameters
NAME          ahmed-samy-CL-N
EXPNO                       2
PROCNO                      1

F2 - Acquisition Parameters
Date_                20231129
Time                    14.00
INSTRUM                 spect
PROBHD      5 mm PABBO BB/
PULPROG                  zg30
TD                      65536
SOLVENT                  MeOD
NS                        128
DS                          2
SWH                  8012.820 Hz
FIDRES               0.122266 Hz
AQ                  4.0894465 sec
RG                      70.92
DW                     62.400 usec
DE                       6.50 usec
TE                      300.0 K
D1                 1.00000000 sec
TD0                         1

======== CHANNEL f1 ========
SFO1              400.1524711 MHz
NUC1                       1H
P1                      12.00 usec
PLW1              18.00000000 W

F2 - Processing parameters
SI                      65536
SF                400.1500000 MHz
WDW                        EM
SSB          0
LB                       0.30 Hz
GB           0
PC                       1.00
```
